# Superstatistical model of bacterial DNA architecture

**DOI:** 10.1038/srep43034

**Published:** 2017-02-22

**Authors:** Mikhail I. Bogachev, Oleg A. Markelov, Airat R. Kayumov, Armin Bunde

**Affiliations:** 1 Biomedical Engineering Research Centre, St. Petersburg Electrotechnical University, St. Petersburg, 197376, Russia; 2 Molecular Genetics of Microorganisms Lab, Institute of Fundamental Medicine and Biology, Kazan (Volga Region) Federal University, Kazan, Tatarstan, 420008, Russia; 3 Institut für Theoretische Physik, Justus-Liebig-Universität Giessen, 35392 Giessen, Germany

## Abstract

Understanding the physical principles that govern the complex DNA structural organization as well as its mechanical and thermodynamical properties is essential for the advancement in both life sciences and genetic engineering. Recently we have discovered that the complex DNA organization is explicitly reflected in the arrangement of nucleotides depicted by the universal power law tailed internucleotide interval distribution that is valid for complete genomes of various prokaryotic and eukaryotic organisms. Here we suggest a superstatistical model that represents a long DNA molecule by a series of consecutive *~*150 bp DNA segments with the alternation of the local nucleotide composition between segments exhibiting long-range correlations. We show that the superstatistical model and the corresponding DNA generation algorithm explicitly reproduce the laws governing the empirical nucleotide arrangement properties of the DNA sequences for various global GC contents and optimal living temperatures. Finally, we discuss the relevance of our model in terms of the DNA mechanical properties. As an outlook, we focus on finding the DNA sequences that encode a given protein while simultaneously reproducing the nucleotide arrangement laws observed from empirical genomes, that may be of interest in the optimization of genetic engineering of long DNA molecules.

The ability of DNA to adopt different structural conformations is a key instrument of many biological processes such as interaction with proteins, replication and transcription. Therefore a better understanding of the DNA mechanical and thermodynamical properties and their impact on its conformational abilities is essential to reveal many regulatory mechanisms at molecular scale and their reflection in the biological systems performance at macroscopic scale. Considerable progress in both theoretical and experimental biophysics in recent decades led to the design and experimental verification of sophisticated mathematical models capable of describing various DNA structural conformations and their physical properties^1^,^2^.

The DNA consists of two complementary polynucleotide chains which form a double helix with a helical pitch of about 10–11 base pairs (bp) that is universal for all kingdoms of life^3^. The primary structure of DNA is determined by a sequence that consists of four nucleotides, namely adenosine (A), cytosine (C), guanosine (G) and thymidine (T). The second polynucleotide chain can be normally reconstructed from the first one due to their complementarity, provided that A is opposed to T and G is opposed to C, and thus statistical analysis can be performed on a single sequence. The two types of base pairs have considerably different bonding energies characterized by the bond enthalpies −11.8 for A:T and −23.8 kcal/mol for G:C, respectively^4^. For an extensive review on the DNA structural organization, we refer to^5^.

Due to its extremely compact packaging, DNA is very efficient as a carrier of genetic information, that is represented by the sequence of nucleotides in its primary structure. Recent success in genetic engineering resulted in the record-breaking amount of information that can be written on synthetic DNA (up to ~200 Mb of data has been reported as of 2016), that is comparable with the entire human genome size, with synthetic DNA patches of thousands to dozens of thousands of base pairs (bp) being already a laboratory routine. One of the key challenges in genetic engineering of long DNA segments is the reproduction of its physical properties such that the synthetic molecule exhibits similar conformational abilities like the DNA in the living cell. The most straightforward approach is to follow the laws that govern the architecture of the host/model organisms and organize the synthetic DNA in a similar way, that in turn requires a better understanding of these laws.

While as a carrier of genetic information the DNA is often treated simply as a sequence of consecutively arranged nucleotides, in fact its molecular structure is much more complex including multiple packaging levels and exhibiting largely heterogeneous properties. From the pure information theory point of view the DNA content is drastically redundant. Even in *Bacteria* where about 97% of DNA encode functional proteins, 20 possible amino acids are being translated from 64 possible trinucleotides that, providing the necessity for the stop codons, already indicates approximately three-fold redundancy. In higher eukaryotes, only about 3% of the DNA encode functional proteins, while up to one half of the remaining 97% non-coding DNA consists of repetitive DNA sequences. The discovery of pronounced long-range correlations (LRC) in DNA sequences in the early 90 s^6^,^7^,^8^,^9^,^10^ only added to this redundancy. LRC in the primary DNA sequence is a signature of clustering of certain nucleotides or nucleotide complexes. In recent years these clustering effects has been utilized in a number of alignment-free bioinformatic approaches via the analysis of the probability distributions of different oligonucleotides in genomes or oligopeptides in corresponding proteomes^11^,^12^,^13^,^14^,^15^,^16^,^17^,^18^,^19^.

Clustering of nucleotides and nucleotide complexes have been attributed to the structural complexity of DNA represented by the famous fractal globule model^20^ that has been recently supported by sequence-based reconstructions of three-dimensional whole-genome architecture^21^,^22^,^23^,^24^,^25^.

Contrast to the multi-level hierarchical eukaryotic DNA architecture, there is a single regular structural level in bacterial DNA, the double helix^3^. At larger scales, it is localized in a relatively free manner in the cytoplasm, with random attachments to the cell membrane and packed in supercoils of varying size. In terms of its mechanical properties, double stranded DNA has a single specific structural scale characterized by its persistence length of about 50 nm, that corresponds to approximately 150 bp^26^,^27^,^28^. The DNA segments displaced by more than persistence length can be considered as no longer mechanically coupled in terms of their tangential directions, and thus structural conformations that are separated by more than 150 bp at a first glance could be treated as independent.

In a recent publication^29^ we have reported a non-trivial universal power law tailed distribution and power law correlations in the internucleotide interval sequences from 130 complete genomes of various organisms from *Archaea* and *Bacteria* to *H. Sapiens* that are valid over many orders of magnitude limited only by the respective genome/chromosome size. This universality is somewhat striking, since it holds for both coding and non-coding, repetitive and non-repetitive DNA segments, and exhibits only moderate variations depending on the GC content of the genomes and on the optimal living temperature of the studied organisms, and thus could be likely attributed to the universal structural and/or conformational properties of DNA that hold independently of the carried genetic information.

The observed universal power law tailed distribution is also known as the *q*-exponential distribution, a subclass of generalized Pareto distributions. In the literature, this class of distributions has been also associated with the maximization of generalized entropy^30^ that had been originally introduced in the early 1960 s by Renyi^31^ and found numerous applications in the analysis of fluctuations in various dynamical systems (see, e.g. ref. 32 and references therein; for the criticism of the concept, see ref. 33 and references therein). In the limit *q* → 1 the *q*-exponential distribution reduces to a simple exponential. In recent years, the same functional form of the distribution have been numerously observed in the dynamical and structural characteristics of several very different complex systems (see, e.g. refs 29, 35, 36, 37, 38 and references therein).

One of the prominent concepts that leads to the observed class of distributions is superstatistics. The superstatistical concept in its original version considers a macroscopic system that consists of microscopic cells exhibiting fluctuations of an intensive quantity, usually denoted as *β*, such as the inverse temperature or dissipation energy^39^,^40^. A local equilibrium is supposed in each of these microscopic cells, while it is achieved at very different *β* values. In this setting, according to the law of total probability, the macroscopic energy distribution is then given by


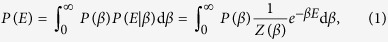


where *P*(*β*) is the distribution of *β* over all local cells in the macroscopic system and *Z*(*β*) is a normalization factor for *e*^−*βE*^ at specified *β*^41^. Under a very general assumption that *β* is additively driven by multiple random factors and each factor is approximately Gaussian distributed, the macroscopic system is described by *q*-exponential distributions^40^,^42^.

In this paper, we take the advantage of the superstatistical approach to suggest a possible explanation of the non-trivial universality in the internucleotide interval distributions. We show explicitly that both the superstatistical model and the corresponding DNA generation algorithm accurately reproduce the laws governing the empirical nucleotide arrangement properties of the bacterial DNA sequences for various GC contents and optimal living temperatures. We also discuss the relevance of our model in terms of DNA mechanical properties. Finally, as an outlook, we focus on finding DNA sequences that encode a given protein while simultaneously reproducing the nucleotide arrangement laws observed from empirical genomes, that may be of interest for the optimization of reverse translation algorithms in the genetic engineering of long DNA molecules.

## Results and Discussion

### Fluctuation analysis

We focus on the same datasets as previously in ref. 29 and start with bacterial DNA that exhibits the simplest structural organization. We split 72 complete bacterial genomes into four groups according to their global GC content determined as the fraction of strongly bonded base pairs (G:C) in the studied genome. For each DNA sequence, we next perform the fluctuation analysis using the widespread detrended fluctuation analysis (DFA) method (for more details on the DFA, we refer to the Methods section at the end of this paper). Since we are mostly interested in the quantities that determine the mechanical properties of DNA and its structural organization, we exchange the nucleotide base pairs by their bond enthalpies, −11.8 for A:T and −23.8 kcal/mol for G:C, respectively^4^.

**Figure 1:**
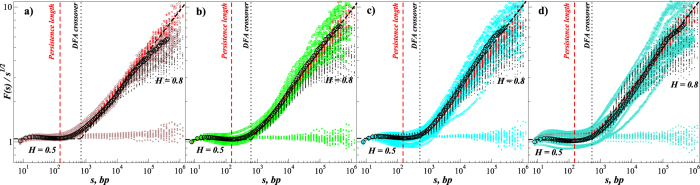
DFA2 fluctuation functions *F*(*s*) for the sequence of base pair bond enthalpies for bacterial genomes with (**a**) very low, (**b**) low, (**c**) intermediate and (**d**) high GC content divided by 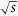 such that a horizontal plateau corresponds to the absence of correlations. Black circles indicate average *F*(*s*) within each group, while dashed lines show the approximate model with effectively vanishing correlations (*H* = 0.5) below and pronounced long-range correlations (*H* = 0.8) above the crossover. Small coloured bubbles show *F*(*s*) for randomly shuffled DNA sequences, while black bubbles show *F*(*s*) for the reconstructed DNA after randomized back-and-forth translation. Corresponding model approximations are given by red *. Vertical dotted lines show the position of the crossover, while the red dashed lines indicate the DNA persistence length.

Our results depicted in Fig. 1 indicate that in all cases *F*(*s*) contains two characteristic regimes, with effectively vanishing correlations below (*H* = 0.5) and pronounced long-range correlations above (*H* = 0.8) the crossover, that is also consistent with the results obtained in earlier studies for other DNA numerical representations^43^,^44^. Taking into account that the second-order DFA used in this study typically shows a characteristic crossover at about 3 … 5 persistence lengths *s*_×_ (see, e.g. ref. 45), the actual position of *s*_×_ at ~150 bp is consistent with the current consensus on the DNA persistence length under physiological conditions widely used in biophysical models (see, e.g. refs [26]). The figure also shows that, as expected, after random shuffling of the nucleotides the correlations vanish at all scales.

### Superstatistical model

**Figure 2:**
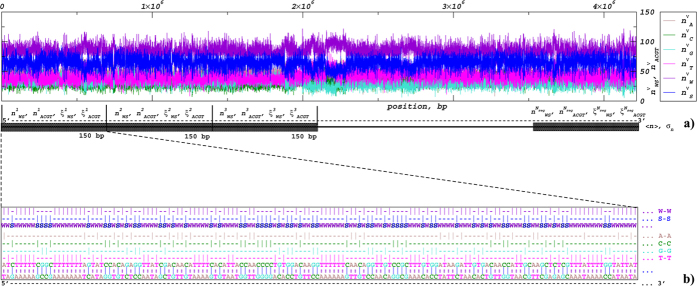
A schematic representation of the superstatistical model and the internucleotide interval sequences assessment procedure exemplified for the *Bacillus subtillis* genomic DNA. (**a**) The DNA sequence is being split into *N*_*seg*_ 150 bp non-overlapping segments *ν* characterized by the local numbers 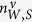 and 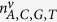 (or their relative fractions 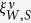 and 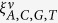, respectively) of weakly ‘W’ or strongly ‘S’ bonded base pairs as well as individual nucleotides ‘A’, ‘C’, ‘G’ and ‘T’, where *ν* runs from 1 to *N*_*seg*_. The upper plot shows the variations of 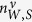 and 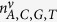 on a genome-size scale. (**b**) An example of 150 bp DNA segment taken from the 5′ end of the *B.subtillis* chromosome is shown in the lower part of the panel, with weakly bonded base pairs connected by dots, while strongly bonded base pairs connected by full vertical lines. Extraction of intervals *l* between consecutive positions of similar nucleotides (A-A, C-C, G-G and T-T) is exemplified for the upstream (5′ to 3′) nucleotide chain. Consecutive occurrences of similar nucleotides (e.g., ‘AA’) is considered as a single internucleotide interval *l* = 1, one nucleotide between similar nucleotides (e.g., ‘ABA’) leads to double interval, where ‘B’ is any nucleotide except ‘A’, and so on. Intervals between consecutive positions of weakly ‘W’ or strongly ‘S’ bonded base pairs are obtained in a similar way from an auxiliary sequence, where either ‘A’ or ‘T’ are exchanged by ‘W’, and either ‘G’ or ‘C’ are exchanged by ‘S’.

Following the above observations, we suggest a simple representation of DNA by a superstatistical model, that is schematically illustrated in Fig. 2. First, we split the DNA sequence into *N*_*seg*_ = [*N*/150[non-overlapping 150 bp segments *ν* = 1, …, *N*_*seg*_, where *N* is the genome size and] …] is the integer operator. We focus on the long-range inter-segment variations of the numbers 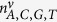 and 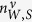 of the respective nucleotides or base pairs per each segment in the analyzed genomes (see Fig. 2a). We also determine the intervals between consecutive positions of similar nucleotides as well as strongly- or weakly bonded base pairs (see Fig. 2b). Due to the effective absence of correlations at short scales (see Fig. 1), we assume that the nucleotides are arranged randomly within each segment, and thus the internucleotide intervals *l* are distributed exponentially *P*(*l*) = 1/ 〈*l*〉exp(−*l*/〈*l*〉), where 〈*l*〉 is the local average interval for each given segment. However, since the numbers of respective nucleotides or base pairs per segment *n* exhibit pronounced segment-to-segment variations along the DNA sequence, this also leads to the variations of the local average interval 〈*l*〉 that is inversely proportional to *n*. In this model, for known *P*(*n*), the marginal distribution is given by


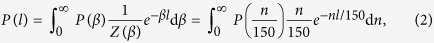


where *β* = *n*/150 is the fraction of specific nucleotides or base pairs in each of the local fragments that plays the role of the local intensity parameter and is bounded between 0 and 1.

**Figure 3:**
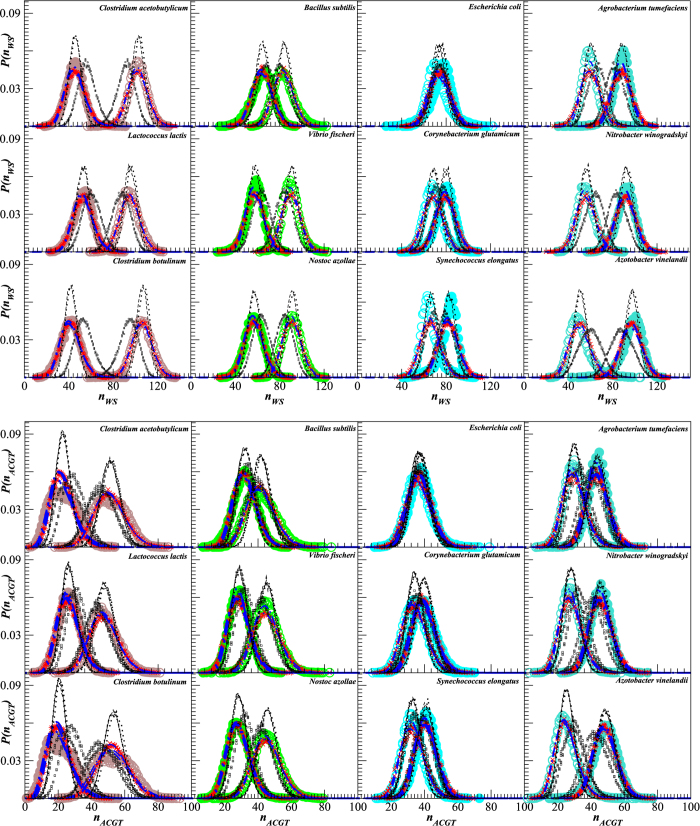
Distribution of the numbers of strongly (•) and weakly (○) bonded base pairs (upper panels) as well as individual nucleotides (lower panels) in the local 150 bp DNA segments for genomes of 12 representative organisms, 3 from each of the very low, low, intermediate and high GC conteof the local numbersnt groups (with GC content increasing from left to right). Corresponding model approximations are given by red *for strongly and by red × for weakly bonded base pairs. Blue dash-dotted lines show model approximations by Γ-distributions. Black dotted and dashed lines show the same distributions for the randomly shuffled DNA sequences and for the randomly generated nucleotide sequences with the same GC content like in the corresponding empirical sequences, respectively. Small black □ symbols show the same distributions for DNA reconstructed from the corresponding proteome after a randomized back-and-forth translation test.

Next we focus on the shape of the distributions *P*(*n*). Figure 3 exemplifies the distributions *P*(*n*) for 12 representative genomes of well-known free living bacteria with different GC contents which are also widely used in molecular biology and genetic engineering. The figure shows that the shapes are very close to Gaussian family distributions, and thus are fully determined by their averages 〈*n*〉 and standard deviations *σ*_*n*_. While a very close approximation could be provided by a Gaussian distribution, it is not physically supported, since it allows for negative values with non-zero probability. Besides a trivial solution such as an effective description by Gaussian truncated at 0 ≤ *n* ≤ 150, similar asymptotic behaviour can be observed in several distributions with a non-negative support such as binomial, Γ- or *χ*^2^-distributions. For large average values 〈*n*〉 the discrepancies between these distributions and Gaussian distributions with corresponding averages 〈*n*〉 and standard deviations *σ*_*n*_ are vanishing. The figure also shows that random shuffling of the nucleotides in the studied DNA sequences leads to considerable narrowing of the distributions, while their shapes remain close to Gaussian.

**Figure 4:**
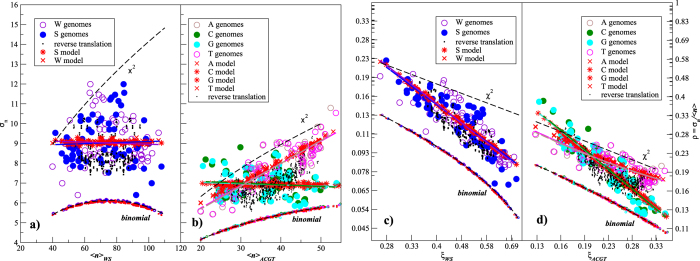
Averages 〈*n*〉 and standard deviations *σ*_*n*_ of the local numbers (**a**) of strongly • or weakly ○ bonded base pairs as well as (**b**) of individual nucleotides A, C, G and T in local 150 bp DNA segments from 72 bacterial genomes. Small coloured symbols show the same quantities for the shuffled DNA sequences as well as for the randomly generated nucleotide sequences with the same GC content like in the empirical data, both following the binomial distributions. Linear regressions are given by full lines, while theoretical combinations of 〈*n*〉 and *σ*_*n*_ for the binomial and *χ*^2^-distributions are given by dashed lines. Corresponding data for the simulated DNA sequences are shown by red * and red × for strongly and weakly bonded nucleotides, respectively. Small black □ symbols show the same data for the DNA sequences obtained by a reverse translation of the corresponding proteomes, as a result of the back-and-forth translation test. (**c,d**) The same data expressed as a relative measure, given by the coefficients of variation *ρ* = *σ*_*n*_/〈*n*〉 as a function of the relative fraction of the given base pairs or nucleotides *ξ*_*WS*_ or *ξ*_*ACGT*_, respectively.

Figure 4a,b shows the parameters 〈*n*〉 and *σ*_*n*_ for the entire set of the studied genomes. Remarkably, *σ*_*n*_ does not depend on the average per segment number over the entire genome 〈*n*〉 for both strongly and weakly bonded base pairs (see Fig. 4a), in contrast to the simple assumptions like the binomial or *χ*^2^-distributions. It is also interesting that the purine-pyrimidine alternation exhibits different properties for strongly and weakly bonded nucleotides (see Fig. 4b). While for G and C *σ*_*n*_ does not change significantly with the changes in their averages per segment 〈*n*〉, for A and T *σ*_*n*_ increases with increasing their averages per segment 〈*n*〉, like in the *χ*^2^ model. Figure 4c,d shows the coefficients of variation *ρ* = *σ*_*n*_/〈*n*〉 as functions of the relative fraction *ξ* =〈*n*〉/150 of given base pairs and individual nucleotides, respectively. The figure shows that in both cases for strongly bonded base pairs and corresponding nucleotides there is approximately algebraical decay *ρ* ∝ 1/*ξ*. The figure also shows that random shuffling of the DNA sequence leads, as expected, to the binomially distributed numbers of strongly- and weakly bonded base pairs as well as individual nucleotides in 150 bp segments.

### DNA simulation algorithm

Next we suggest an simulation algorithm which generates a DNA sequence with random genetic code where the local number of nucleotides within 150 bp segments exhibits similar distributions and long-range correlation properties, like in the empirical bacterial genomes.

In the first step, we generate a long-range correlated dataset 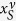 with *H* = 0.8 with zero mean and unit variance consisting of *N*_*seg*_ numbers. To determine the number of strongly bonded base pairs 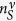 in each segment *ν*, we next multiply 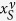 by the standard deviation *σ*_*n*_ = 9 that corresponds to the average standard deviation observed from empirical genomes and add the mean 〈*n*_*s*_〉 equal to the average number of strongly bonded base pairs per segment of the simulated genome.

In the second step, a simulated DNA sequence is created consisting of *N*_*seg*_ consecutive segments, with *ν*^th^ segment containing 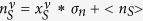 strongly bonded base pairs ‘S’ that are randomly allocated within the segment. The remaining 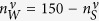 positions are filled with weakly bonded base pairs ‘W’.

In the third step, the positions of strongly- and weakly bonded base pairs are filled by either purines (A or G) or pyrimidines (C or T) in the primary polypeptide chain by exchanging ‘W’ by either ‘A’ or ‘T’, and ‘S’ by either ‘G’ or ‘C’. For that, two other long-range correlated dataset with *H* = 0.8 consisting of *N*_*seg*_ numbers each that are independent of the first dataset are created. One of these datasets gives the probabilities 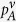 that a purine ‘A’ replaces ‘W’ for each segment *ν*. Accordingly, the number of ‘A’ in segment *ν* is given by 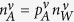, and the number of ‘T’ is given by 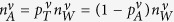. We found that the mean value 〈*p*_*A*_〉 = 0.5 and standard deviation 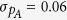 leads to the best agreement with the empirical data, independently of both local and global GC contents.

In contrast, for strongly bonded base pairs, ‘S’ is exchanged by purine ‘G’ with probabilities taken from the third long-range correlated dataset with mean 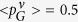, but now with standard deviation 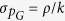, where *ρ* = *σ*_*n*_/〈*n*〉 is the coefficient of variation of the average number of strongly bonded base pairs per segment that is adjusted according to the global GC content of the studied genome, and *k* = 1.75 is the empirically adjusted correction coefficient. After that the remaining ‘S’ are exchanged by ‘C’. In all cases, particular positions of either purines or pyrimidines within segments are chosen randomly, due to vanishing short-range correlations.

We have simulated 72 datasets each corresponding to a single bacterial genome in terms of its size and global GC content. Figure 4 indicates that the simulated DNA follows the regression lines that indicate the representative *σ*_*n*_ and *ρ* for the given 〈*n*〉 and *ξ*, respectively. Indeed, a more specific adjustment to the particular host/model organism genome properties is possible by taking its specific *σ*_*n*_ instead of the universal *σ*_*n*_ = 9 and fitting a specific empirical coefficient *k* for the purine-pyrimidine alternation procedure from empirical genome data.

Nevertheless, Fig. 3 shows that the distributions of the numbers of given nucleotides or base pairs in local 150 bp segments can be well reproduced by the superstatistical model and by the DNA simulation algorithm despite of using not exact, but typical *σ*_*n*_ values for the given GC content. To emphasize the reproducibility of our results for a larger set of bacterial genomes, more data are shown in the Supplementary information (available) as Figs S1 and S2 for very low, Figs S5 and S6 for low, Figs S9 and S10 for intermediate as well as Figs S13 and S14 for high overall GC content.

### Internucleotide interval distributions

Since neither binomial nor *χ*^2^ distributions fit the combinations of 〈*n*〉 and *σ*_*n*_ observed in the empirical data, we next follow a more generalized model with a positive support that could represent *P*(*n*), and thus also the relative quantity *P*(*β*) for all considered cases, that is the Γ-distribution


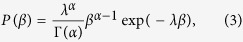


where *β* = *n*/150 plays the role of the local intensity parameter, Γ(*α*) is the Γ-function, *α* is the shape parameter and *λ* is the rate parameter. The average of the Γ-distribution is given by 〈*n*〉 = *α*/*λ* and its variance equals 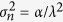. Accordingly, the shape parameter can be expressed as *α* = 1/(*σ*_*n*_/〈*n*〉)^2^ = 1/*ρ*^2^ such that it depends only on the coefficient of variation *ρ* = *σ*_*n*_/〈*n*〉, and the rate parameter *λ* = *α*/〈*n*〉 adjusts for the average number of a given nucleotide per 150 bp segment 〈*n*〉. Figure 3 shows that Γ-distributions with above parameters provide reasonable quality approximations despite of using not exact, but typical *σ*_*n*_ values for the given GC content.

For the entire DNA molecule, we have the superposition of multiple DNA segments with exponential internucleotide interval distributions characterized by local averages 〈*l*〉 that are inversely proportional to the local numbers of nucleotides *n*, that in turn are proportional to the local “intensity parameter” *β*, and thus the overall interval distribution for the entire genome can be determined in the framework of the above superstatistical concept. Following Eq. 1 with Γ-distributed *P*(*β*) one easily obtains


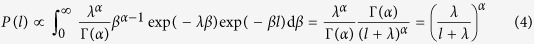


that is asymptotically equivalent to the *q*-exponential distribution *P*(*l*)∝(1 + (*q* − 1)(*α*/*λ*)*l*)^−1/(*q*−1)^ = (1 + (*q* − 1) 〈*n*〉*l*)^−1/(*q*−1)^, where *q* = 1/(1 + *α*) = 1 + (*σ*_*n*_/〈*n*〉)^2^ = 1 + *ρ*^2^ determines the slope of the asymptotic power law behaviour and 〈*n*〉 adjusts the position of the crossover, in agreement with our previous empirical findings^29^. Accordingly, the observational power law tailed distribution can be obtained directly from the law of total probability and also can be expressed in a simpler form than the *q*-exponential. Furthermore, it seems likely that the reason for the wide occurrence of *q*-exponentials in complex systems is simply the superposition of some locally independent patterns with different local concentrations, that can be explained by similar superstatistical models.

**Figure 5:**
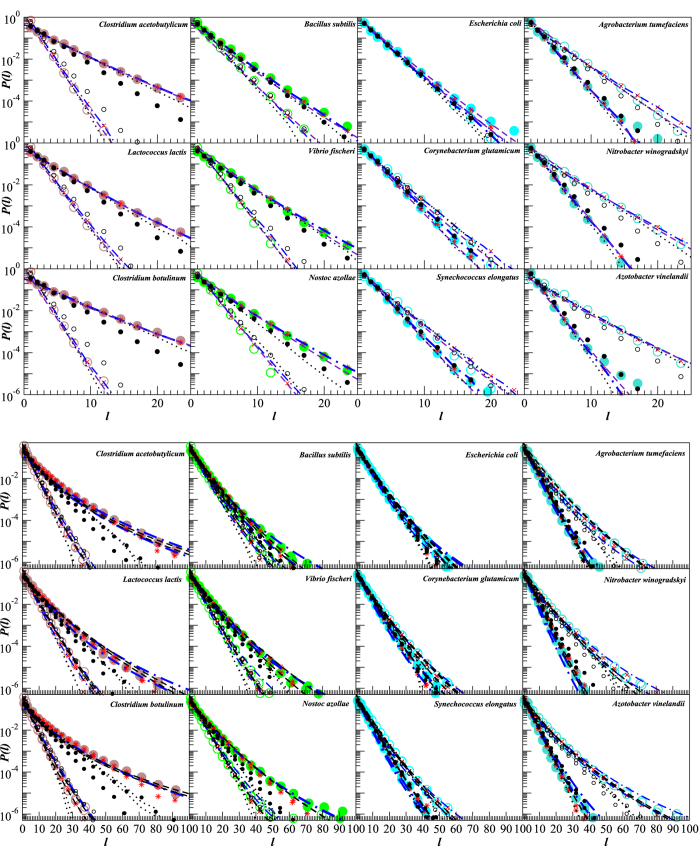
Distribution of the intervals between strongly (coloured •) and weakly (coloured ○) bonded base pairs (upper panels) as well as between individual nucleotides (lower panels) for genomes of 12 representative organisms, 3 from each of the very low, low, intermediate and high GC content groups (with GC content increasing from left to right). Corresponding model approximations are given by red * for strongly and by red × for weakly bonded base pairs. Blue dash-dotted lines show results of numerical integration according to Eq. 2, while black dashed lines show corresponding approximations by *q*-exponential distributions. Dotted lines show the same distributions for the randomly shuffled DNA sequences. Small black • symbols show the same distributions after the randomized back-and-forth translation test.

Figure 5 shows that the internucleotide interval distributions are also quite well reproduced by both the superstatistical model as well as by the corresponding distributions in the simulated DNA. The fitted distributions exhibit the same values of *α* and *λ* parameters as predicted by the above analytical treatment. In particular, for the distributions of intervals between strongly or weakly bonded base pairs, *α* decreases from *α* ≈ 200 to *α* ≈ 20 with increasing the coefficient of variation *ρ* from 0.07 to 0.23, that corresponds to decreasing *ξ* from *ξ* ≈ 0.7 to *ξ* ≈ 0.3 (see also outliers in Fig. 4). For the distributions of intervals between G and C nucleotides, *α* increases from *α* ≈ 6 to *α* ≈ 50 for *ξ* increasing from *ξ* ≈ 0.13 to *ξ* ≈ 0.35, while for the distributions of intervals between G and C nucleotides, *α* increases from *α* ≈ 10 to *α* ≈ 30 for *ξ* increasing from *ξ* ≈ 0.13 to *ξ* ≈ 0.35, due to their more narrow range of the coefficients of variation *ρ* (see also Fig. 4). The figure also shows that random shuffling of the DNA sequence, as expected, leads to the reduction of the internucleotide interval distributions to simple exponentials that can be observed as straight lines in the semi-logarithmic plots. To emphasize the reproducibility of our results for a larger set of bacterial genomes, more internucleotide interval distributions and their model predictions are shown in the Supplementary information (available) as Figs S3 and S4 for very low, Figs S7 and S8 for low, Figs S11 and S12 for intermediate as well as Figs S15 and S16 for high overall GC content.

### Back-and-forth translation test

Next we simulated translations of each studied genome to obtain corresponding proteomes, and then a reverse translation to obtain a DNA sequence that encodes the given proteome. While the first procedure is straightforward and unambiguous, the second one is less trivial due to the inherent redundancy of the genetic code. We used a reverse translation procedure that randomly selects one of the triplets corresponding to a given amino acid residue. Taking into account that well above 90% of the bacterial genome contains coding DNA, there are only minor differences between the sizes of the original and the back-and-forth translated datasets, and thus corresponding finite size effects should be comparable. Despite that, as one can clearly observe from the above figures, there are significant discrepancies between the distributions of the numbers of given nucleotides in local 150 bp segments as well as between the distributions of the internucleotide intervals for the original and back-and-forth translated DNA. Deviations are especially pronounced when the GC content is significantly different from 50%. This indicates that the random choice of triplets corresponding to a given amino acid disrupts the variations of the local average numbers 〈*n*〉 and fractions *ξ* of the strongly- and weakly bonded base pairs as well as individual nucleotides and, especially, the long-range correlations in the genome, leading to the significant changes in the fluctuation functions (see Fig. 1) as well as to the corruption of the internucleotide interval distributions (see Fig. 5). Moreover, the randomized back-and-forth translation test leads to the distributions that are considerably further away from the empirical data than similar distributions for the randomly shuffled DNA sequences. This is easy to explain, since random shuffling preserves the total number and thus also the fractions of nucleotides, while back-and-forth translation does not. More sophisticated reverse translation algorithms are often adjusted to fit the global properties of the given host/model organism such as its GC content or the fraction of each codon in the genome, which only partially resolves the problem, since the long-range correlations are nevertheless corrupted.

### The effects of optimal living temperature and extreme living conditions

In a recent study^29^ we have observed moderate discrepancies between the internucleotide interval distributions in organisms with different optimal living temperatures that might be a sign of their adaptation to the external conditions. In terms of genome statistics, an easily observed sign of adaptation to non-typical thermodynamical constraints are specific laws governing the variation of both global and local GC contents. We next verify the validity of the suggested model for a cohort of extremophile organisms belonging to the *Archaea* kingdom. Remarkably, among three groups consisting of genomes from organisms with optimal living temperatures below 50 °C, between 50 °C and 80 °C and above 80 °C, the first and the last groups have comparable average GC contents^29^.

Supplementary Figure S17 (available) shows the fluctuation functions *F(s)* for the studied archaeal genomes. The figure shows that there are significant variations of the crossover position both in the normal and high living temperature groups. While it has been shown that DNA persistence length decreases with increasing temperature^28^, there might be additional effects arising from the adaptation to other extreme living factors such as high salinity and high pressure.

In the Supplementary information (available) similar distributions as in Fig. 3 for extremophile *Archaea* can be observed. In particular, Figs S18 and S19, Figs S22 and S23 as well as Figs S26 and S27 (available) show the corresponding distributions for the organisms with optimal living temperatures below 50 °C, between 50 °C and 80 °C and above 80 °C, respectively. The figures show that in several observed cases there are considerable deviations between the empirical and the model distributions, indicating that direct extrapolation of the laws governing the local arrangement of nucleotides in free living *Bacteria* does not always work for other microbial organisms such as extremophile *Archaea*. Distribution asymmetry, skewness and other shape modifications such as flattening of the distribution indicated by a plateau in its central part can be observed in several examples from different groups of extremophile genomes.

Supplementary Figs S20 and S21, Figs S24 and S25 as well as Figs S28 and S29 (available) show the intervals between strongly and weakly bonded base pairs as well as between individual nucleotides in the genomes of extremophile *Archaea* with optimal living temperatures below 50 °C, between 50 °C and 80 °C and above 80 °C, respectively. The figures show that, while the results of numerical integration according to Eq. 2 leading to the power law tailed approximations according to Eq. 4 agree with the empirical internucleotide interval distributions to a certain extent, the DNA simulation algorithm in several cases fails to reproduce the asymptotic behaviour of the distributions indicating that the nucleotide arrangement differs from that one observed in *Bacteria*, and only an effective phenomenological description is possible.

### Hierarchical model, or superstatistics of superstatistics

Contrast to bacterial DNA, eukaryotic genomes exhibit complex multi-level hierarchical structural organization. In a recent publication^29^, we reported that in the genomes of higher eukaryotes including humans internucleotide intervals exhibit a complex two-compound distribution that could be well approximated by two additive *q*-exponentials with *q* ≈ 1.11, that is asymptotically equivalent to the power law tailed distributions of form (4) with *α* ≈ 9. Supplementary Figure S30 (available) shows that such a distribution can be to a certain extent reproduced in a simulated DNA sequence that is organized as a hierarchical cascade. The first level, like in the bacterial DNA model, contains 150 bp segments, that for eukaryotes is also close to another important characteristic scale, the 146 bp cycle of DNA wrapping around a histone^5^. Next, 150 segments are organized in a single supersegment, and an additional long-range correlated variability parameter with the same *H* = 0.8 is added for these larger 150^2^ bp supersegments, that are now of the common size of chromatin loops. Finally, a third-level variability parameter also with *H* = 0.8 is added for 150^3^ bp supersupersegments that correspond to the typical size of isochores, large fragments of eukaryotic genomes that are characterized by stable GC content. By tuning the weights for these large-scale variability parameters, one can obtain a distribution that exhibits similar shape like in the empirical genomes of higher eukaryotes.

Indeed, the approximate reproduction of the distribution of internucleotide intervals cannot guarantee that the simulated DNA sequence would correspond to the same spatial structure as the empirical DNA exhibits. The eukaryotic DNA architecture has seven known characteristic scales, which should be implemented explicitly in a proper model setting to reproduce its spatial organization. However, we believe that similar principles based on superstatistical approach could be applied to characterize also the large-scale organization of eukaryotic DNA, and the internucleotide interval distributions could be useful as a testbed to verify the implementation of the respective nucleosome packaging and chromatin positioning models.

### Predicting DNA mechanical properties

The suggested model could be also used for the statistical prediction of some mechanical properties of long DNA strands. It is well known that particular composition of nucleotides determines the local DNA curvature^46^, bending energy and several other important mechanical properties. In particular, at short scales the local sub-elastic chain (LSEC)^47^,^48^ and at long scales the worm-like chain (WLC) models^49^,^50^ accurately predict the local bending energy, that is proportional to the linear and squared local cumulative bending angles for given DNA segments, respectively^51^. The suggested superstatistical model can be easily extended to describe the corresponding DNA curvature or bending energy distributions by considering the local cumulative bending angle Θ as the local intensity parameter *β* and to obtain the respective macroscopic curvature or energy distributions from Eq. 1. In the absence of particular nucleotide composition, representative statistics for a DNA fragment of given size and GC content could be obtained by using the representative model parameters that have been used above to simulate the DNA sequences.

### Adahptation of synthetic DNA patches to the host organism architecture: An outlook towards potential implications for genetic engineering

**Figure 6:**
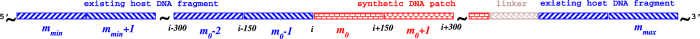
A schematic representation of the synthetic DNA patch insertion into existing host DNA. Existing part of host DNA upstream of the synthetic DNA patch is split into 150 bp segments *m*_0_ − 1, *m*_0_ − 2, …, *m*_*min*_ and is used to calculate the optimized local concentrations *ξ*_*WS*_ and *ξ*_*ACGT*_ in the synthetic DNA patch segments *m*_0_, *m*_0_ + 1 and so on, according to Eq. 5. Further downstream the synthetic patch is connected with the remaining part of the host DNA (with or without an optional linker) in the way that the local concentrations *ξ*_*WS*_ and *ξ*_*ACGT*_ in following DNA fragments *m* until *m*_*max*_ also follow Eq. 5.

In most practical scenarios, a synthetic genetic construction has to be inserted at a specific position *i* into the DNA of the host organism (see Fig. 6). The simplest case is when only one of the synthetic DNA strands carries a gene, e.g., in the direction from 5′ to 3′. Our goal is to suggest the synthetic insertion that would appear like a natural extrapolation of the preceding DNA of the host/model organism. For that, we first analyze the preceding host DNA by splitting it into 150 bp non-overlapping segments at positions *i* − 150, *i* − 300 and so on and calculating the local fractions of strongly and weakly based pairs *ξ*_*WS*_ as well as the local fractions of individual nucleotides *ξ*_*A*,*C*,*G*,*T*_ in each segment. Next we use our suggested model to predict the local fractions of both strongly/weakly bonded based pairs as well as individual nucleotides by the optimal extrapolation of the same quantities from the preceding host DNA onwards to the synthetic fragment following ref. 52


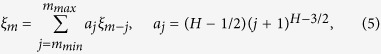


where *m* is the current segment number that changes between *m*_*min*_ and *m*_*max*_ spanning over both the preceding piece of host DNA and the synthetic DNA patch. The calculation starts from 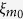 that corresponds to the first segment *m*_0_ in the synthetic DNA patch, while the data used for calculation considers also the fractions of strongly/weakly bonded base pairs in the preceding segments of the host DNA by taking into account also *j* < *m*_0_. The prediction is continued iteratively for 150 bp segments of the synthetic DNA patch up to its completion. After the local fractions of both strongly/weakly based pairs as well as of individual nucleotides are obtained for each 150 bp segment, they can be used as target quantities for the reverse translation optimization. During the reverse translation procedure, the current fractions of strongly/weakly bonded base pairs are calculated from already back-translated codons. In each ambiguous case, when a single amino acid residue can be represented by several codons, one should always choose that codon that leads to the local fraction of strongly/weakly bonded base pairs characterized by the minimum square displacement from the prediction provided by the model for the current 150 bp segment. Next, the same procedure is repeated for the purine-pyrimidine alternation, separately for choosing between G and C in strongly and between A and T in weakly bonded base pairs, in each case taking the respective model parameters, this way resolving the remaining ambiguity.

Finally, similar optimization strategy can be employed to find the best ending position of the synthetic DNA patch including, if necessary, an optimal size and content of the linker between the synthetic patch and the forward piece of host DNA. If the synthetic DNA patch has to encode functional genes on each of its strands, an optimization problem with two equations for the directions from 5′ to 3′ and from 3′ to 5′ similar to Eq. 5 arises, and a best fit can be found iteratively.

## Conclusion

To summarize, we have suggested a superstatistical model that is capable of reproducing the statistical laws that govern the arrangement of nucleotides in the primary sequence of bacterial DNA. The model considers the entire DNA molecule as a concatenation of non-overlapping segments, with each segment characterized by its own number (or fraction) of strongly/weakly bonded base pairs as well as individual nucleotides. The size of the segments is determined by the DNA persistence length that is approximately 150 bp under physiological conditions. We assume that within each segment the nucleotides are allocated randomly, corresponding to the local equilibrium scenario, while the numbers (or fractions) of different nucleotides in consecutive segments alternate in a long-range correlated manner with *H* = 0.8. Based on this model, we have also suggested a DNA simulation procedure that explicitly reproduces the typical distributions of internucleotide intervals for bacterial genomes with very different GC contents. We have also shown that the predictions by the model can to a certain extent reproduce similar properties in other microbial genomes, while some deviations can be observed in several extremophile *Archaea*, that might be attributed to their adaptation to the extreme living conditions such as high temperatures, pressure and salinity, that also affects the DNA persistence length. We also show that the suggested superstatistical model can be organized in a hierarchical cascade, that could lead to a reasonable reproduction of the internucleotide interval distributions in higher eukaryotic genomes, and thus might be also useful as a testbed for the respective nucleosome packaging and chromatin positioning models. Finally, as an outlook, we suggest how the proposed model could be utilized to facilitate the adaptation of the synthetic genetic constructions by choosing the most appropriate codons that would lead to the best reproduction of the empirical laws governing the nucleotide arrangement at particular positions in the genomes of the respective host/model organisms, that is an important issue for genetic engineering of long DNA patches.

## Methods

### Detrended fluctuation analysis

The detrended fluctuation analysis (DFA) method was originally suggested by Peng *et al*. and generalized by Kantelhardt *et al*. and Hu *et al*.^8^,^45^,^53^. It is based on the analysis of the profile, or the cumulative sum 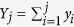 of the raw series of numbers *y*_*i*_. The profile is split into *N*_*s*_ non-overlapping segments of size *s*. In each segment *k* one determines the best polynomial fit *y*_*k*_(*j*) and obtains the variance 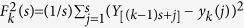 between the local trend and the profile in each segment *k*. Finally, one obtains the fluctuation function *F*(*s*) by averaging over all segments


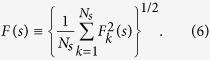


For long-range correlated data *F*(*s*) scales with *s* as *F*(*s*)~*s*^*H*^, where *H* is the Hurst exponent directly related to the autocorrelation function *C*(*s*) by *C*(*s*)~*s*^−*γ*^, where *γ* = 2 − 2*H*. When the correlations are vanishing, *C*(*s*) = 0 for *s* > 0, and *H* = 1/2.

Fluctuation analysis deals with sequences of numbers, not nucleotides, and thus in general the results depend on the way how the studied DNA sequence has been replaced by a numeric sequence. Early studies focused mainly on the replacement of a given nucleotide (A, C, G or T) or their combination by one, while the others by zero^54^, or on the “DNA walks” which increase by one when a pyrimidine (C or T) is observed and decrease by one when a purine (A or G) is observed in a DNA sequence^7^. Since we are mostly interested in the properties that determine the mechanical and thermodynamical properties of DNA, we exchange the nucleotide base pairs by their bond enthalpies, −11.8 for A:T and −23.8 kcal/mol for G:C, respectively^4^.

### Interval distributions

Distributions of intervals between similar items in a series are known to explicitly reflect the persistence properties of data sequences. If the items of interest are allocated randomly, intervals between them follow a simple exponential distribution 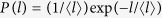, where 〈*l*〉 is the average interval, and are uncorrelated. In linearly long-range correlated data, one expects the asymptotic PDF of the intervals to follow a stretched exponential 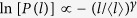, with exponent *γ* = 2 − 2*H*^55^,^56^, where *H* is the Hurst exponent characterizing the LRC. In the presence of nonlinear correlations, the PDF gets even broader and decays asymptotically by a power-law *P*(*l*)~(*l*/〈*l*〉)^−*δ*^, where the exponent *δ* decreases when the LRC gets more pronounced^57^,^58^. Related analytical results on gap sizes and cluster sizes for widespread models including random walks, Levy flights as well as for systems exhibiting phase transitions have been obtained^59^,^60^,^61^. In recent years, interval distributions have been shown to efficiently reflect structural and dynamical features of complex systems in physics, biology, geoscience, climate, finance and many other applications^35^,^36^,^37^,^55^,^56^,^57^,^58^,^62^. Besides already mentioned application to the primary structure of DNA^29^, very recently an approach to the prediction of structural, localization and functional properties of unknown proteins based on their explicit reflection in the distributions of intervals between similar amino acid residues has been suggested^63^.

## Additional Information

**How to cite this article:** Bogachev, M. I. *et al*. Superstatistical model of bacterial DNA architecture. *Sci. Rep.* **7**, 43034; doi: 10.1038/srep43034 (2017).

**Publisher's note:** Springer Nature remains neutral with regard to jurisdictional claims in published maps and institutional affiliations.

## Supplementary Material

Supplementary Information
