# Corrigendum: Superstatistical model of bacterial DNA architecture

**DOI:** 10.1038/srep46917

**Published:** 2017-12-22

**Authors:** Mikhail I. Bogachev, Oleg A. Markelov, Airat R. Kayumov, Armin Bunde

Scientific Reports 7: Article number: 43034; 10.1038/srep43034 published online: 02 22 2017; updated: 12 22 2017.

This Article contains errors. An improper normalization factor was inadvertently applied, resulting in an incorrect form of Eq. (4). In the Introduction section,

“In this setting, according to the law of total probability, the macroscopic energy distribution is then given by
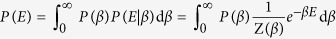
where P(*β*) is the distribution of *β* over all local cells in the macroscopic system and Z(*β*) is a normalization factor for e^−*βE*^ at specified *β*^41^.”

should read:

“In this setting, according to the law of total probability, the macroscopic energy distribution is then given by
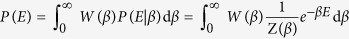
where W(*β*) is the distribution of *β* over all local cells in the macroscopic system and Z(*β*) is a normalization factor for e^*−βE*^ at specified *β*^41^.”

Therefore in the Results and Discussion section under the subheading ‘Superstatistical model’,

“Due to the effective absence of correlations at short scales (see Fig. 1), we assume that the nucleotides are arranged randomly within each segment, and thus the internucleotide intervals *l* are distributed exponentially *P*(*l*) = 1/〈*l*〉 exp(−*l*/〈*l*〉), where 〈*l*〉 is the local average interval for each given segment. However, since the numbers of respective nucleotides or base pairs per segment *n* exhibit pronounced segment-to-segment variations along the DNA sequence, this also leads to the variations of the local average interval 〈*l*〉 that is inversely proportional to *n*. In this model, for known *P*(*n*), the marginal distribution is given by
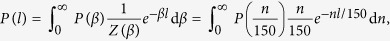
where *β* = *n*/150 is the fraction of specific nucleotides or base pairs in each of the local fragments that plays the role of the local intensity parameter and is bounded between 0 and 1.

Next we focus on the shape of the distributions *P*(*n*).”

should read:

“Due to the effective absence of correlations at short scales (see Fig. 1), we assume that in each of the *N*_seg_ segments of length 150 bp we have *n*_*ν*_ randomly located sites, where *n*_*ν*_ could stand for the number of S,W,A,C,G or T in segment *ν*. Then in the *ν*-th segment, the mean interval length 〈*l*_*ν*_〉 = 150/*n*_*ν*_ and the probability *P*_*ν*_(*l*) of finding an interval of length *l* is given by
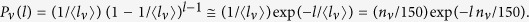


Accordingly, the probability *P*(*l*) of finding an interval of length *l* in all *N*_seg_segments is given by
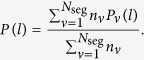
By introducing *β*_*ν*_ = *n*_*ν*_/150 that specifies the local fraction of the considered sites in segment *ν*, *P*(*l*) becomes
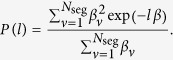
By introducing the probability density *W*(*β*) the sum over the segments can be replaced by an integral,
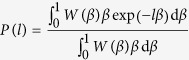
By substituting 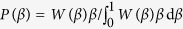 we finally obtain
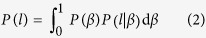
in agreement with the law of total probability (1).

Next we focus on the shape of the distributions *P*(*β*). For simplicity, we study the related distributions of the numbers *n* of sites in each fragment *P*(*n*) = *P*(150 · *β*)/150. “

In addition, under the subheading “Internucleotide interval distributions”,

“Since neither binomial nor *χ*^2^ distributions fit the combinations of 〈*n*〉and *σ*_*n*_ observed in the empirical data, we next follow a more generalized model with a positive support that could represent *P*(*n*), and thus also the relative quantity *P*(*β*) for all considered cases, that is the Γ-distribution
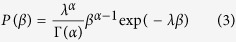
where *β* = *n*/150 plays the role of the local intensity parameter, Γ(*α*) is the Γ-function, *α* is the shape parameter and *λ* is the rate parameter.”

should read:

“Since neither binomial nor *χ*^2^ distributions fit the combinations of 〈*n*〉 and *σ*_*n*_ observed in the empirical data, we next follow a more generalized model with a positive support that could represent *P*(*n*) for all considered cases, that is the Γ-distribution
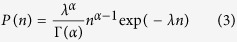
where *β* = *n*/150 plays the role of the local intensity parameter, Γ(*α*) is the Γ-function, *α* is the shape parameter and *λ* is the rate parameter.”

Furthermore,

“Following Eq. (1) with Γ-distributed *P* (*β*) one easily obtains
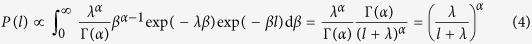
that is asymptotically equivalent to the q-exponential distribution.


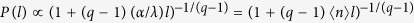
, where 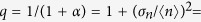 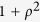 determines the slope of the asymptotic power law behaviour and 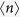 adjusts the position of the crossover, in agreement with our previous empirical findings^29^.”

should read:

“Accordingly, for Γ-distributed *P*(*n*) the local fractions *W*(*β*) are also Γ-distributed as 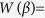 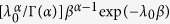, with 〈*β*〉 = *α*/*λ*_0_ and 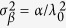, where the shape parameter *α* is the same as for *P*(*n*), while the rate parameter *λ*_0_ = 150 · *λ*. Then *P*(*β*) is also Γ-distributed with the shape parameter (*α* + 1), and thus the marginal distribution of intervals follows
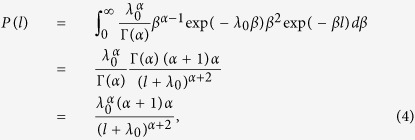
that is equivalent to the *q*-exponential distribution 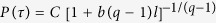 with 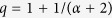, 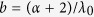 and 
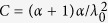
, in agreement with our previous empirical findings^29^.

This discrepancy, while providing different asymptotic behavior, can hardly be observed for real bacterial DNA sequences. The reason is that 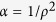 is inversely proportional to the squared coefficient of variation 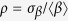 of the fractions of given sites *β*. Since under normal conditions long DNA segments with nearly no strongly bonded base pairs cannot exist over long time due to their instability, and long DNA segments with nearly solely strongly bonded base pairs would require irrelevantly high energy consumption for unwinding that precede replication and transcription thereby lacking its function, we always remain at 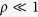. This leads to 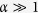 thus making the decay rates of *α* and *α* + 2 hardly distinguishable in observational plots. Our tests indicate that within the available range of *P*(*l*) up to the maximum internucleotide interval *l* that can be observed in studied bacterial DNA sequences this correction does not change significantly the PDF shapes and affects neither the validity of the figures of *P*(*l*) in the original version of this Article nor the conclusions that have been drawn specifically for the bacterial DNA. However, this correction may appear important when modeling considerably longer (e.g. eukaryotic) DNA sequences as well as other complex systems where similar laws can be observed with 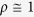,with exponentially distributed intensity parameter 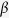 as a prominent example.

