# Supplementary material for: Superstatistical model of bacterial DNA architecture
Source: Sci Rep. 2017 Feb 22;7:43034. doi: 10.1038/srep43034 (PMC5320525; doi:10.1038/srep43034)
Supplement: Supplementary Information [file srep43034-s1.pdf]

Supplementary information to the article "Superstatistical model of bacterial DNA architecture"

Mikhail I. Bogachev, Oleg A. Markelov, Airat R. Kayumov, Armin Bunde

January 12, 2017

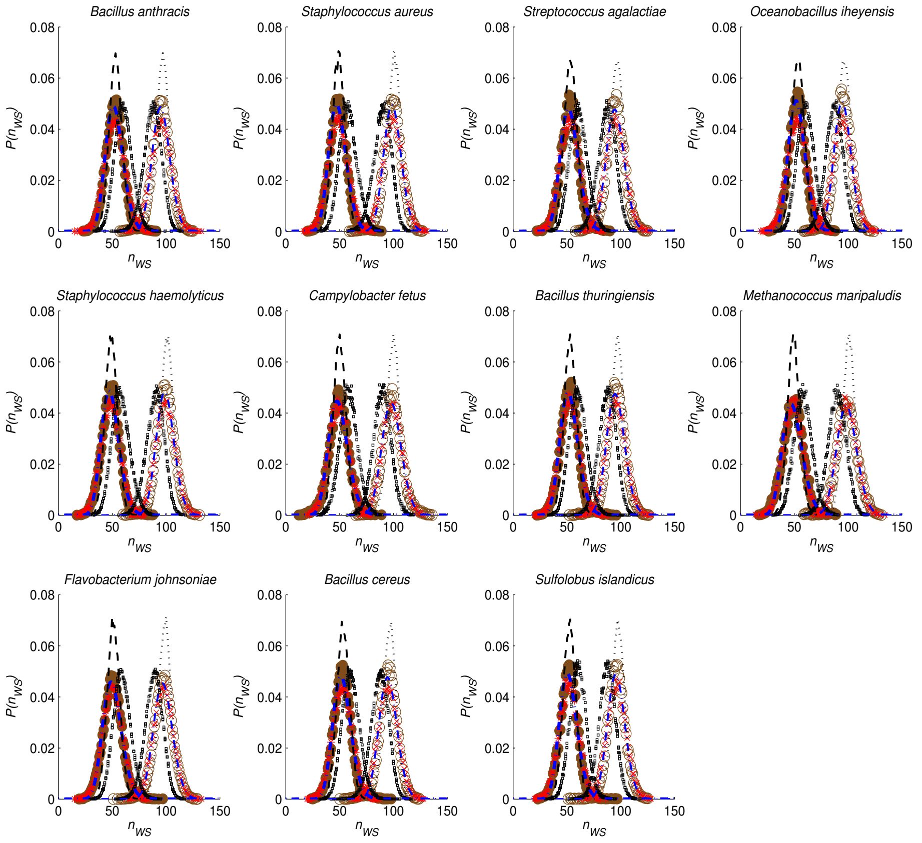

Figure S1: Distribution of the numbers of strongly (•) and weakly (∘) bonded base pairs in the local 150 bp DNA segments for bacterial genomes with very low GC content. Corresponding model approximations are given by red $*$ for strongly and by red $\times$ for weakly bonded base pairs. Blue dash-dotted lines show model approximations by Γ-distributions. Black dashed and dotted lines show the same distributions for the randomly shuffled DNA sequences for strongly and weakly bonded base pairs, respectively. Small black $\Box$ symbols show the same distributions for DNA reconstructed from the corresponding proteome after a randomized back-and-forth translation test.

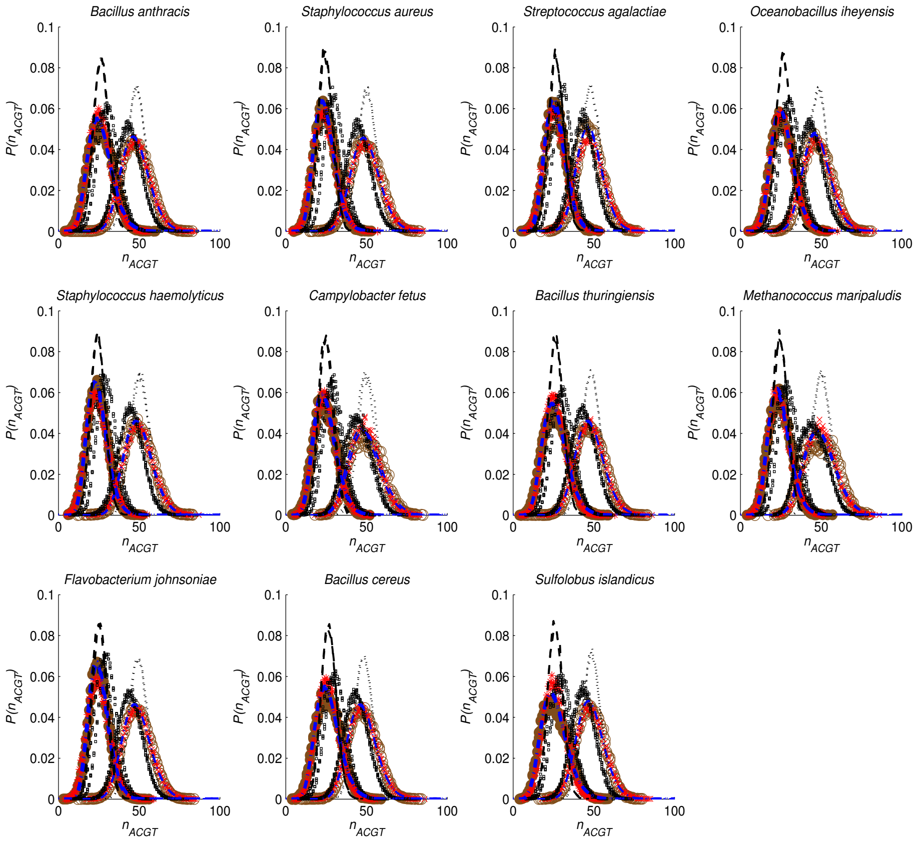

Figure S2: Distribution of the numbers of strongly (•) and weakly (∘) bonded nucleotides in the local 150 bp DNA segments for bacterial genomes with very low GC content. Corresponding model approximations are given by red $*$ for strongly and by red $\times$ for weakly bonded nucleotides. Blue dash-dotted lines show model approximations by Γ-distributions. Black dashed and dotted lines show the same distributions for the randomly shuffled DNA sequences for strongly and weakly bonded nucleotides, respectively. Small black □ symbols show the same distributions for DNA reconstructed from the corresponding proteome after a randomized back-and-forth translation test.

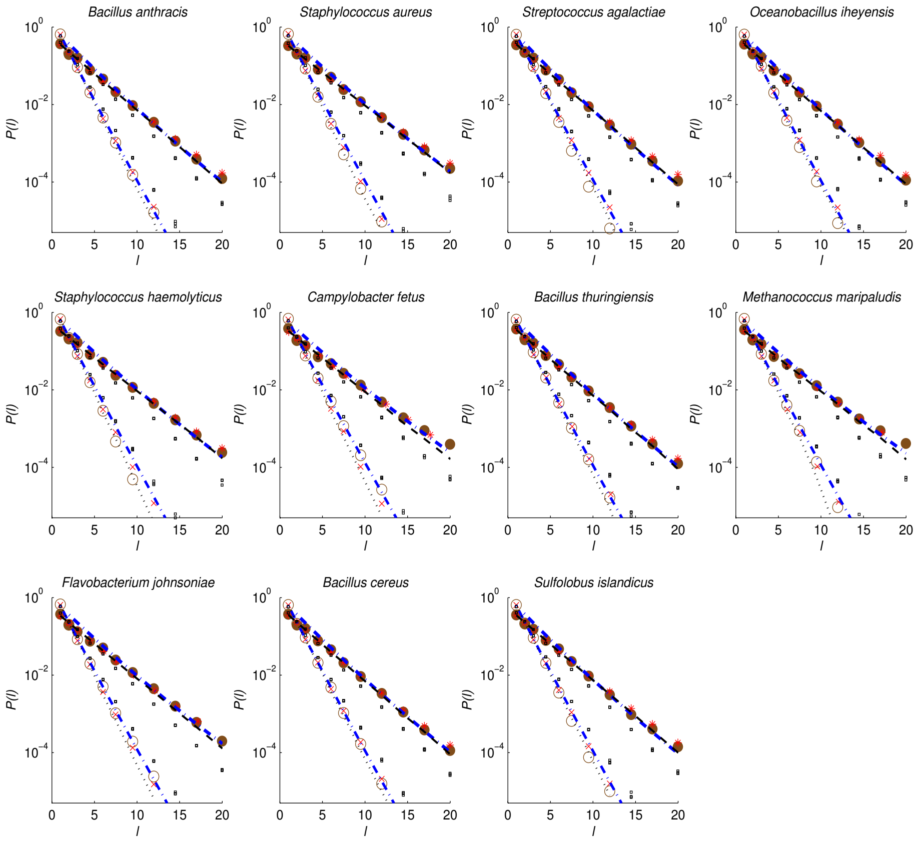

Figure S3: Distribution of the intervals between strongly (•) and weakly (◦) bonded base pairs for bacterial genomes with very low GC content. Corresponding model approximations are given by red $*$ for strongly and by red $\times$ for weakly bonded base pairs. Blue dash-dotted lines show corresponding approximations by power law tailed distributions according to Eq. 4. Black dashed and dotted lines show the same distributions for the randomly shuffled DNA sequences for strongly and weakly bonded base pairs, respectively. Small black □ symbols show the same distributions for DNA reconstructed from the corresponding proteome after a randomized back-and-forth translation test.

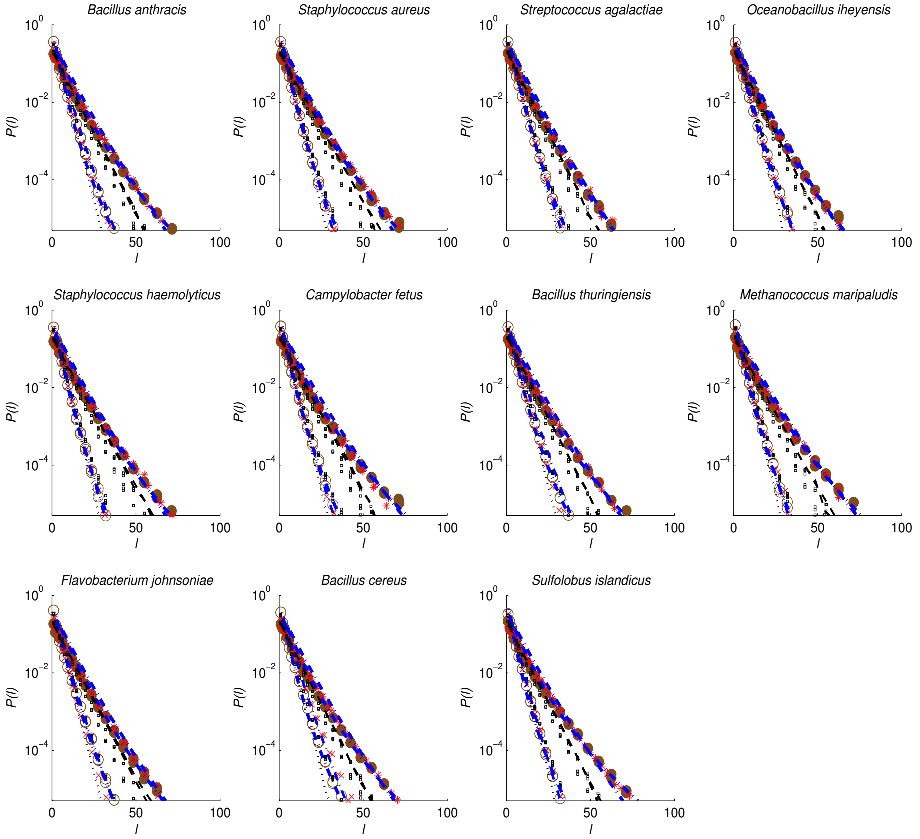

Figure S4: Distribution of the intervals between strongly (•) and weakly (◦) bonded nucleotides for bacterial genomes with very low GC content. Corresponding model approximations are given by red $*$ for strongly and by red $\times$ for weakly bonded base pairs. Blue dash-dotted lines show corresponding approximations by power law tailed distributions according to Eq. 4. Black dashed and dotted lines show the same distributions for the randomly shuffled DNA sequences for strongly and weakly bonded nucleotides, respectively. Small black □ symbols show the same distributions for DNA reconstructed from the corresponding proteome after a randomized back-and-forth translation test.

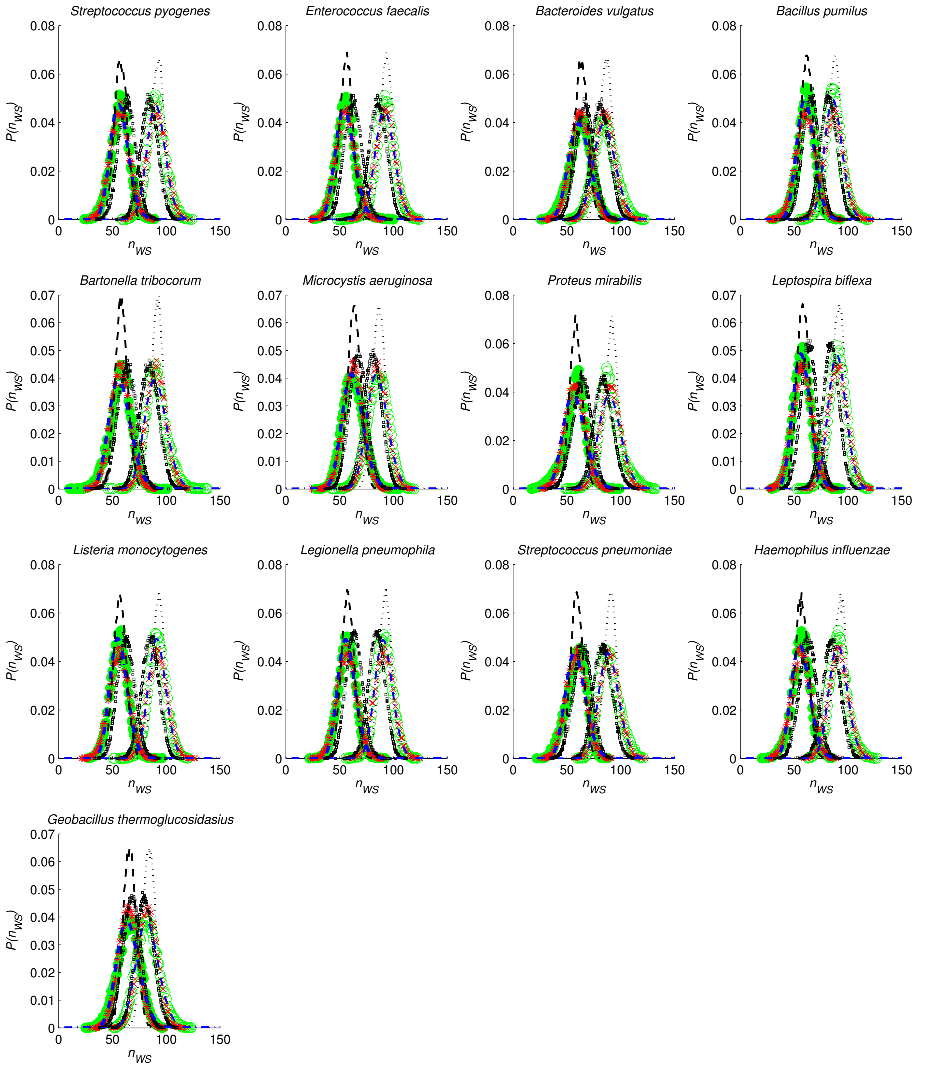

Figure S5: Distribution of the numbers of strongly (•) and weakly (◦) bonded base pairs in the local 150 bp DNA segments for bacterial genomes with low GC content. Corresponding model approximations are given by red $*$ for strongly and by red $\times$ for weakly bonded base pairs. Blue dash-dotted lines show model approximations by Γ-distributions. Black dashed and dotted lines show the same distributions for the randomly shuffled DNA sequences for strongly and weakly bonded base pairs, respectively. Small black $\square$ symbols show the same distributions for DNA reconstructed from the corresponding proteome after a randomized back-and-forth translation test.

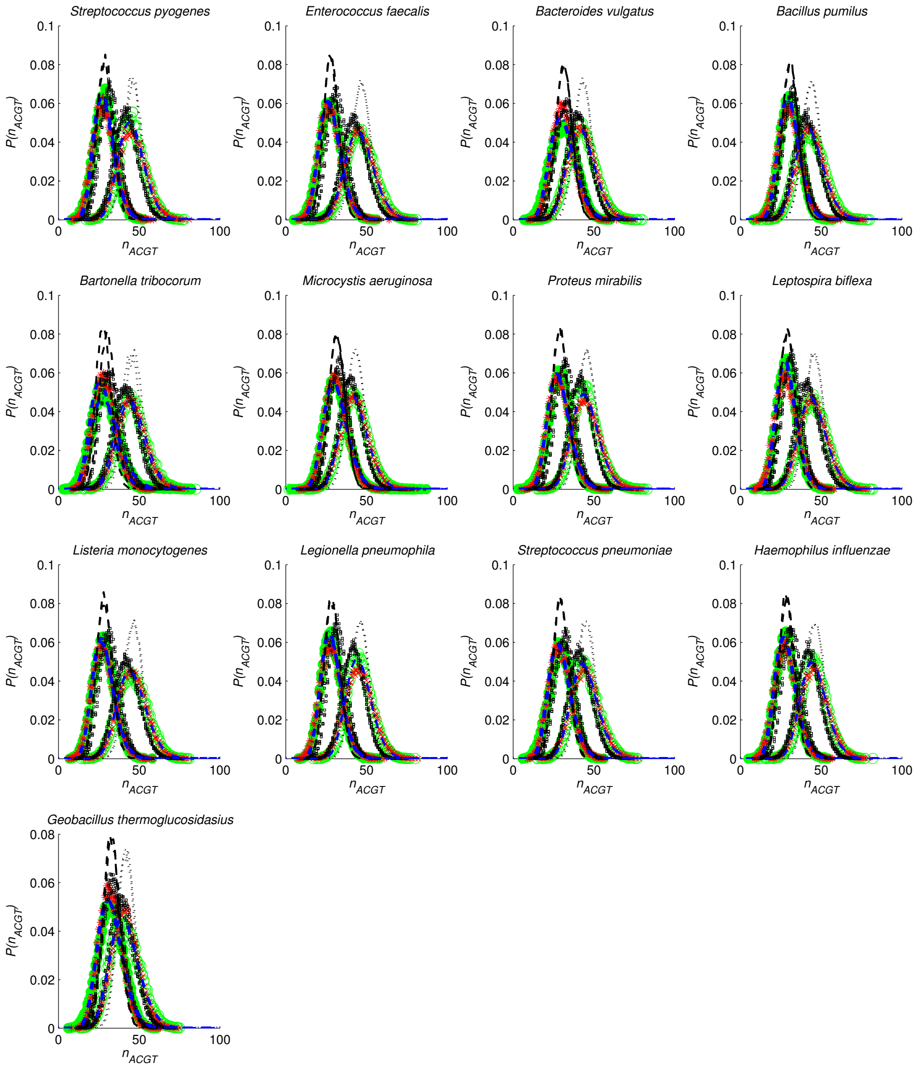

Figure S6: Distribution of the numbers of strongly (•) and weakly (◦) bonded nucleotides in the local 150 bp DNA segments for bacterial genomes with low GC content. Corresponding model approximations are given by red $*$ for strongly and by red $\times$ for weakly bonded nucleotides. Blue dash-dotted lines show model approximations by Γ-distributions. Black dashed and dotted lines show the same distributions for the randomly shuffled DNA sequences for strongly and weakly nucleotides pairs, respectively. Small black □ symbols show the same distributions for DNA reconstructed from the corresponding proteome after a randomized back-and-forth translation test.

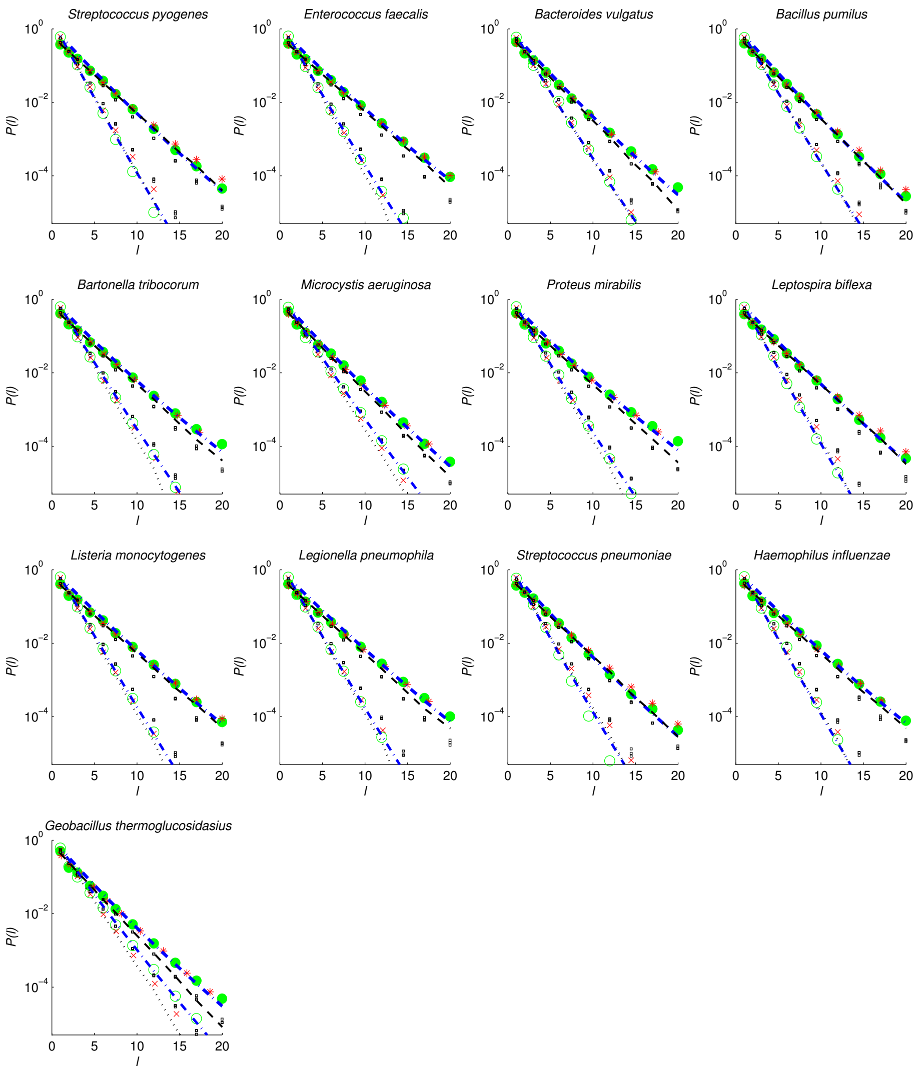

Figure S7: Distribution of the intervals between strongly (•) and weakly (◦) bonded base pairs for bacterial genomes with low GC content. Corresponding model approximations are given by red $*$ for strongly and by red $\times$ for weakly bonded base pairs. Blue dash-dotted lines show corresponding approximations by power law tailed distributions according to Eq. 4. Black dashed and dotted lines show the same distributions for the randomly shuffled DNA sequences for strongly and weakly bonded base pairs, respectively. Small black □ symbols show the same distributions for DNA reconstructed from the corresponding proteome after a randomized back-and-forth translation test.

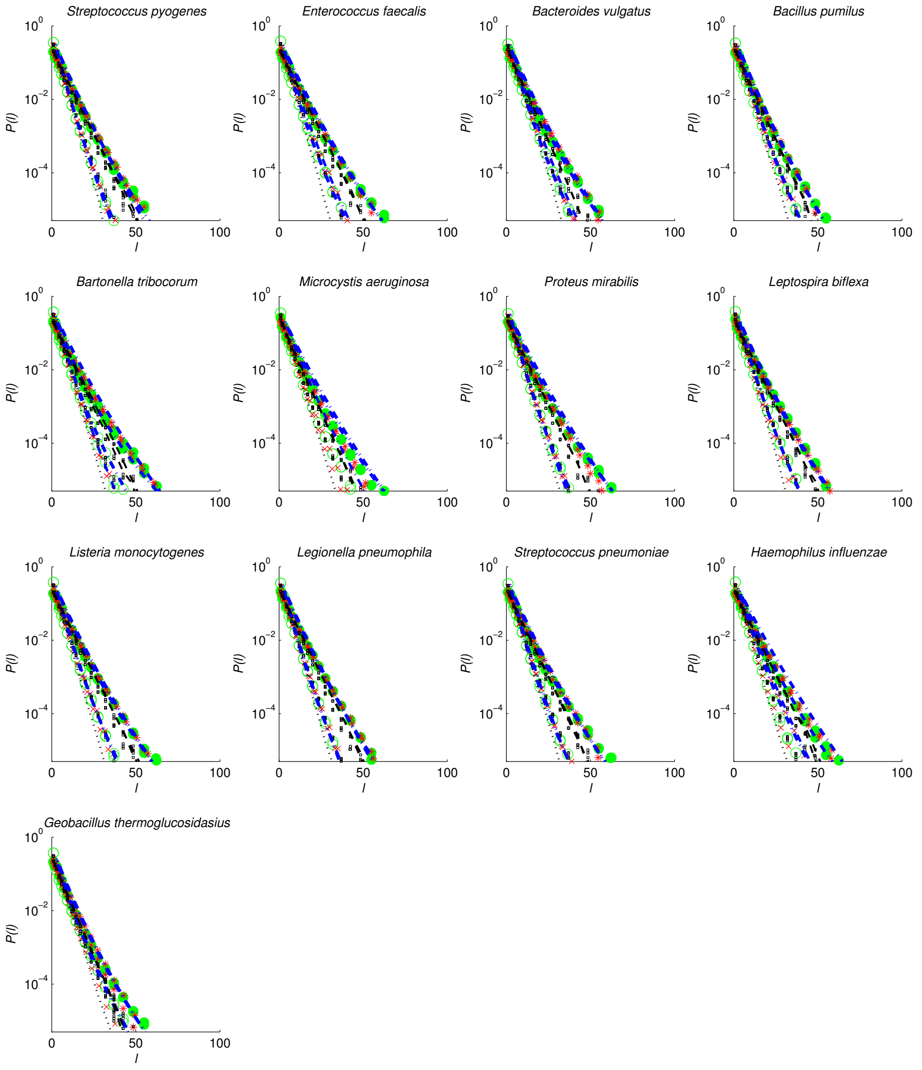

Figure S8: Distribution of the intervals between strongly (•) and weakly (○) bonded nucleotides for bacterial genomes with low GC content. Corresponding model approximations are given by red $*$ for strongly and by red $\times$ for weakly bonded base pairs. Blue dash-dotted lines show corresponding approximations by power law tailed distributions according to Eq. 4. Black dashed and dotted lines show the same distributions for the randomly shuffled DNA sequences for strongly and weakly bonded nucleotides, respectively. Small black □ symbols show the same distributions for DNA reconstructed from the corresponding proteome after a randomized back-and-forth translation test.

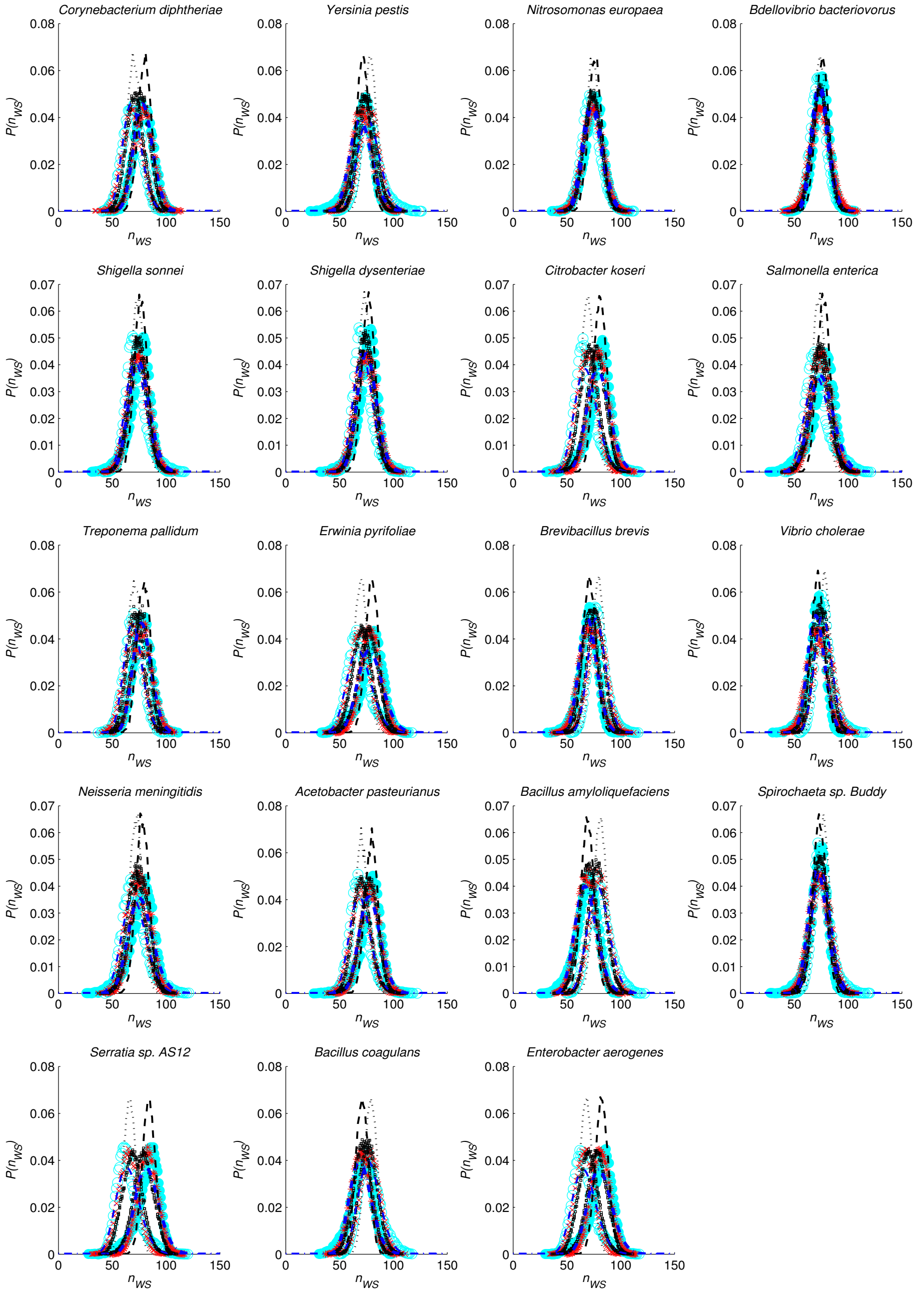

Figure S9: Distribution of the numbers of strongly (•) and weakly (◦) bonded base pairs in the local 150 bp DNA segments for bacterial genomes with intermediate GC content. Corresponding model approximations are given by red $*$ for strongly and by red $\times$ for weakly bonded base pairs. Blue dash-dotted lines show model approximations by Γ-distributions. Black dashed and dotted lines show the same distributions for the randomly shuffled DNA sequences for strongly and weakly bonded base pairs, respectively. Small black $\square$ symbols show the same distributions for DNA reconstructed from the corresponding proteome after a randomized back-and-forth translation test.

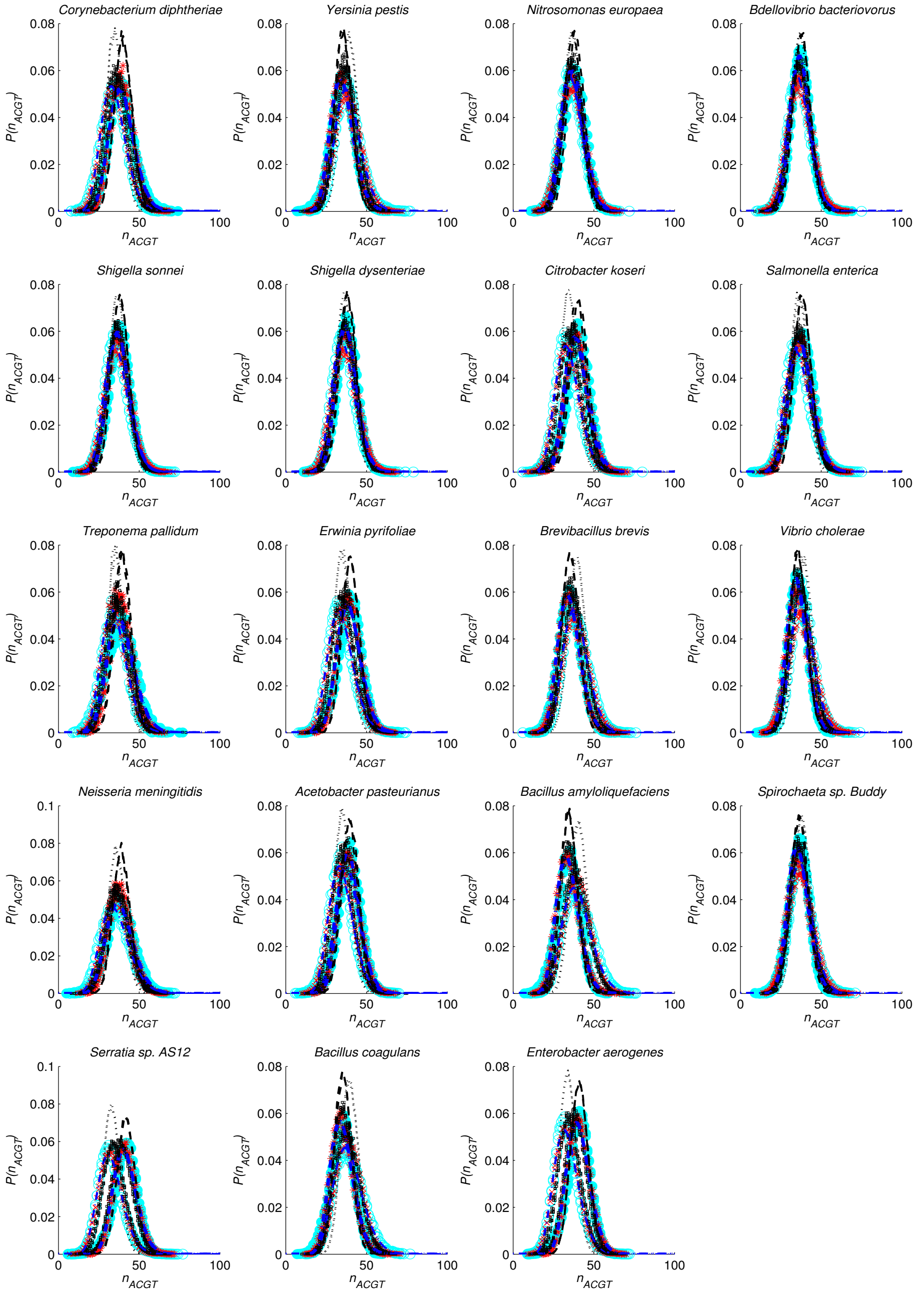

Figure S10: Distribution of the numbers of strongly (•) and weakly (∘) bonded nucleotides in the local 150 bp DNA segments for bacterial genomes with intermediate GC content. Corresponding model approximations are given by red $*$ for strongly and by red $\times$ for weakly bonded nucleotides. Blue dash-dotted lines show model approximations by Γ-distributions. Black dashed and dotted lines show the same distributions for the randomly shuffled DNA sequences for strongly and weakly nucleotides pairs, respectively. Small black □ symbols show the same distributions for DNA reconstructed from the corresponding proteome after a randomized back-and-forth translation test.

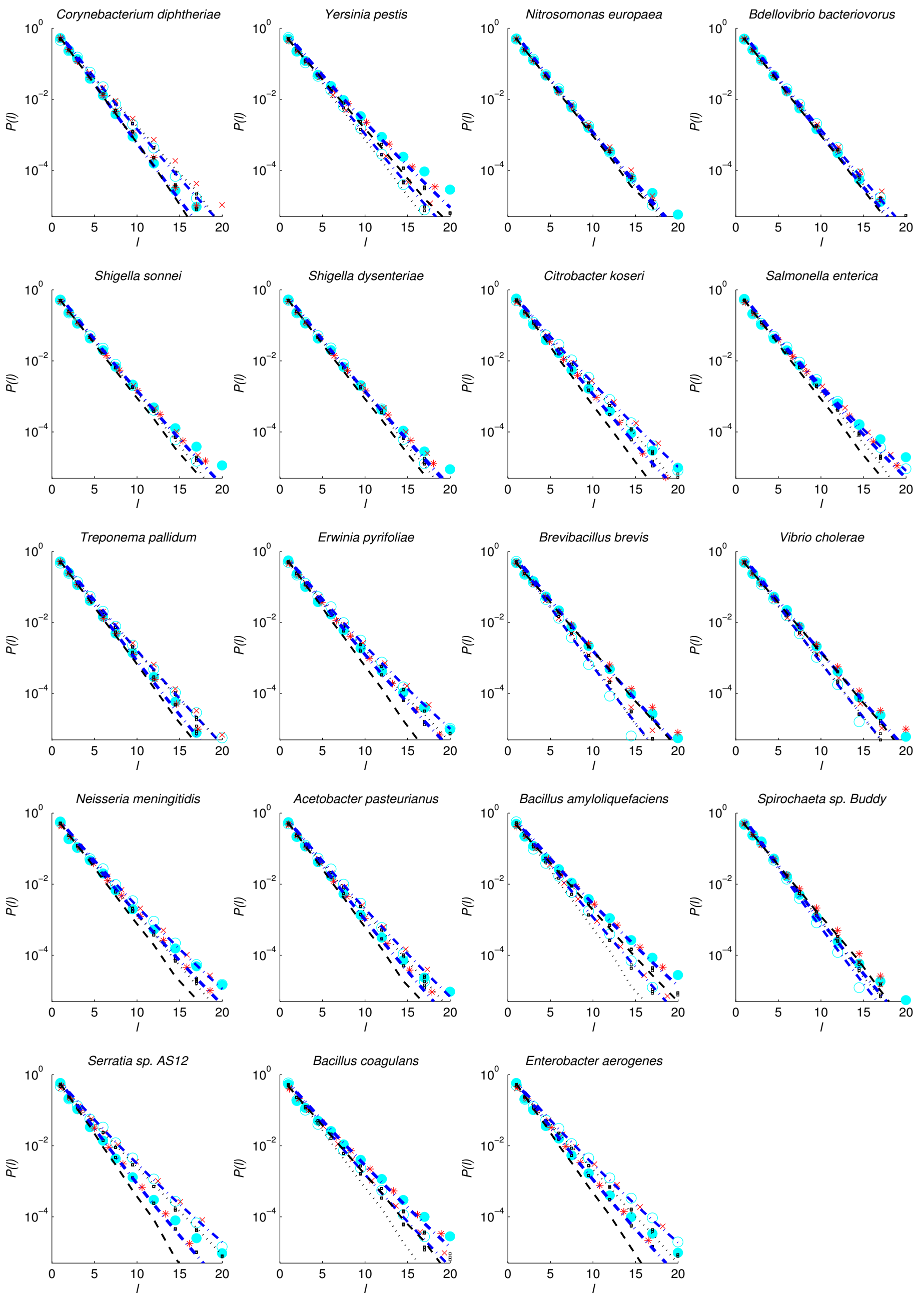

Figure S11: Distribution of the intervals between strongly (•) and weakly (◦) bonded base pairs for bacterial genomes with intermediate GC content. Corresponding model approximations are given by red ∗ for strongly and by red × for weakly bonded base pairs. Blue dash-dotted lines show corresponding approximations by power law tailed distributions according to Eq. 4. Black dashed and dotted lines show the same distributions for the randomly shuffled DNA sequences for strongly and weakly bonded base pairs, respectively. Small black □ symbols show the same distributions for DNA reconstructed from the corresponding proteome after a randomized back-and-forth translation test.

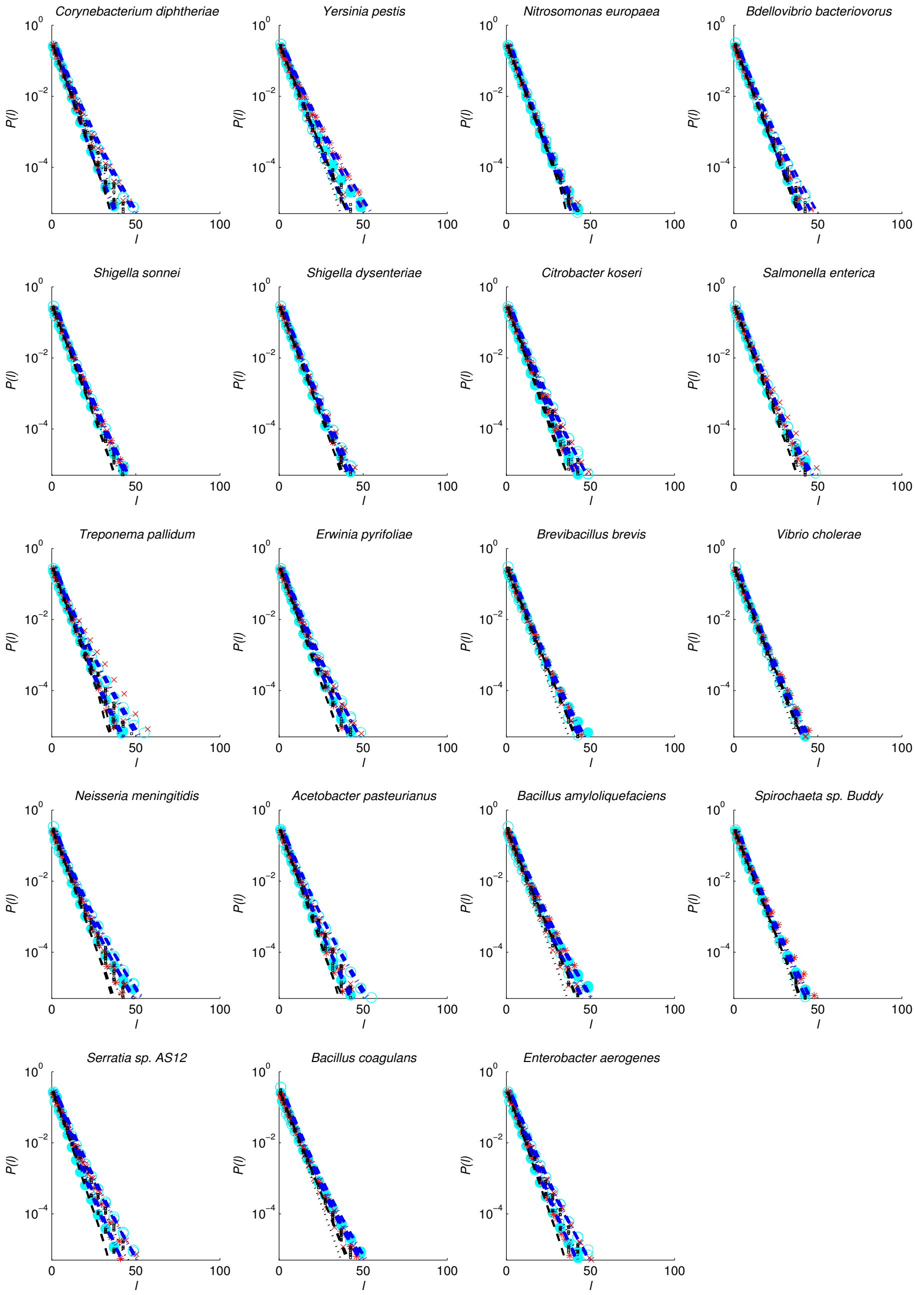

Figure S12: Distribution of the intervals between strongly (•) and weakly (∘) bonded nucleotides for bacterial genomes with intermediate GC content. Corresponding model approximations are given by red $*$ for strongly and by red $\times$ for weakly bonded base pairs. Blue dash-dotted lines show corresponding approximations by power law tailed distributions according to Eq. 4. Black dashed and dotted lines show the same distributions for the randomly shuffled DNA sequences for strongly and weakly bonded nucleotides, respectively. Small black □ symbols show the same distributions for DNA reconstructed from the corresponding proteome after a randomized back-and-forth translation test.

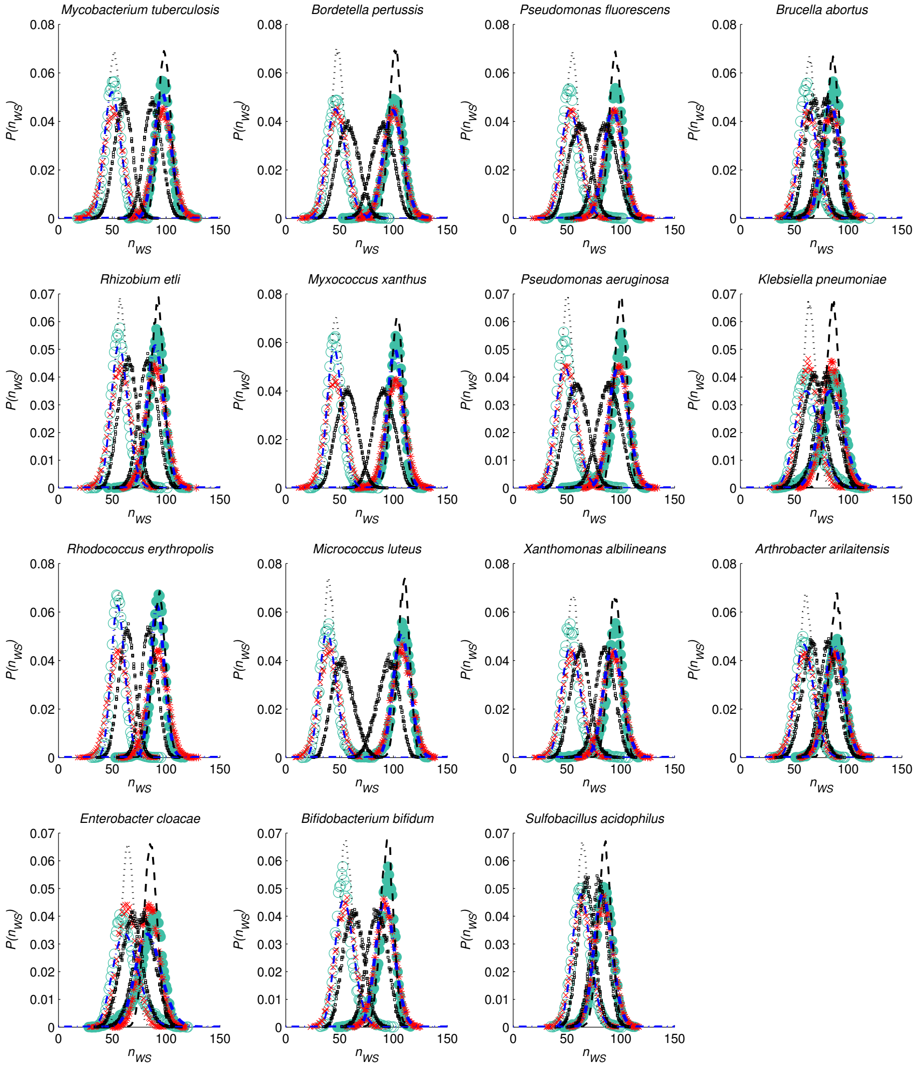

Figure S13: Distribution of the numbers of strongly (•) and weakly (◦) bonded base pairs in the local 150 bp DNA segments for bacterial genomes with high GC content. Corresponding model approximations are given by red $*$ for strongly and by red $\times$ for weakly bonded base pairs. Blue dash-dotted lines show model approximations by Γ-distributions. Black dashed and dotted lines show the same distributions for the randomly shuffled DNA sequences for strongly and weakly bonded base pairs, respectively. Small black □ symbols show the same distributions for DNA reconstructed from the corresponding proteome after a randomized back-and-forth translation test.

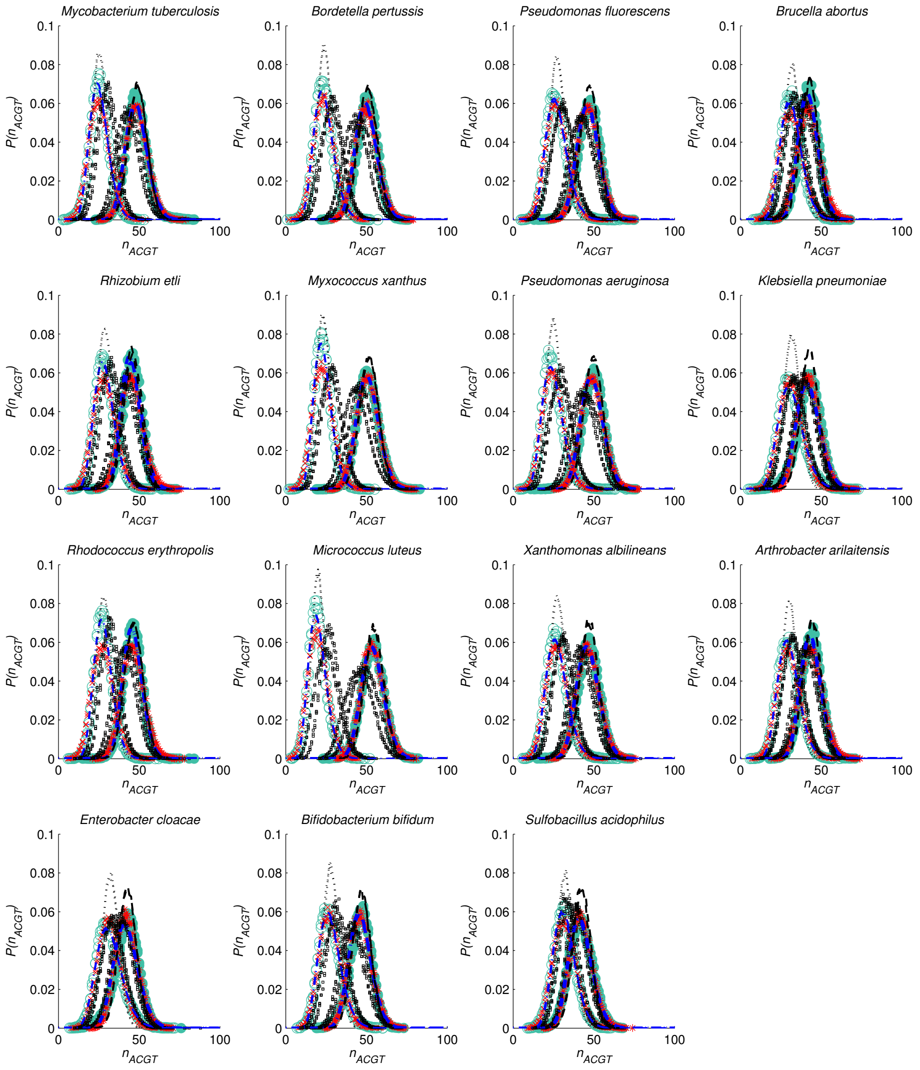

Figure S14: Distribution of the numbers of strongly (•) and weakly (∘) bonded nucleotides in the local 150 bp DNA segments for bacterial genomes with high GC content. Corresponding model approximations are given by red ∗ for strongly and by red × for weakly bonded nucleotides. Blue dash-dotted lines show model approximations by Γ-distributions. Black dashed and dotted lines show the same distributions for the randomly shuffled DNA sequences for strongly and weakly bonded nucleotides, respectively. Small black □ symbols show the same distributions for DNA reconstructed from the corresponding proteome after a randomized back-and-forth translation test.

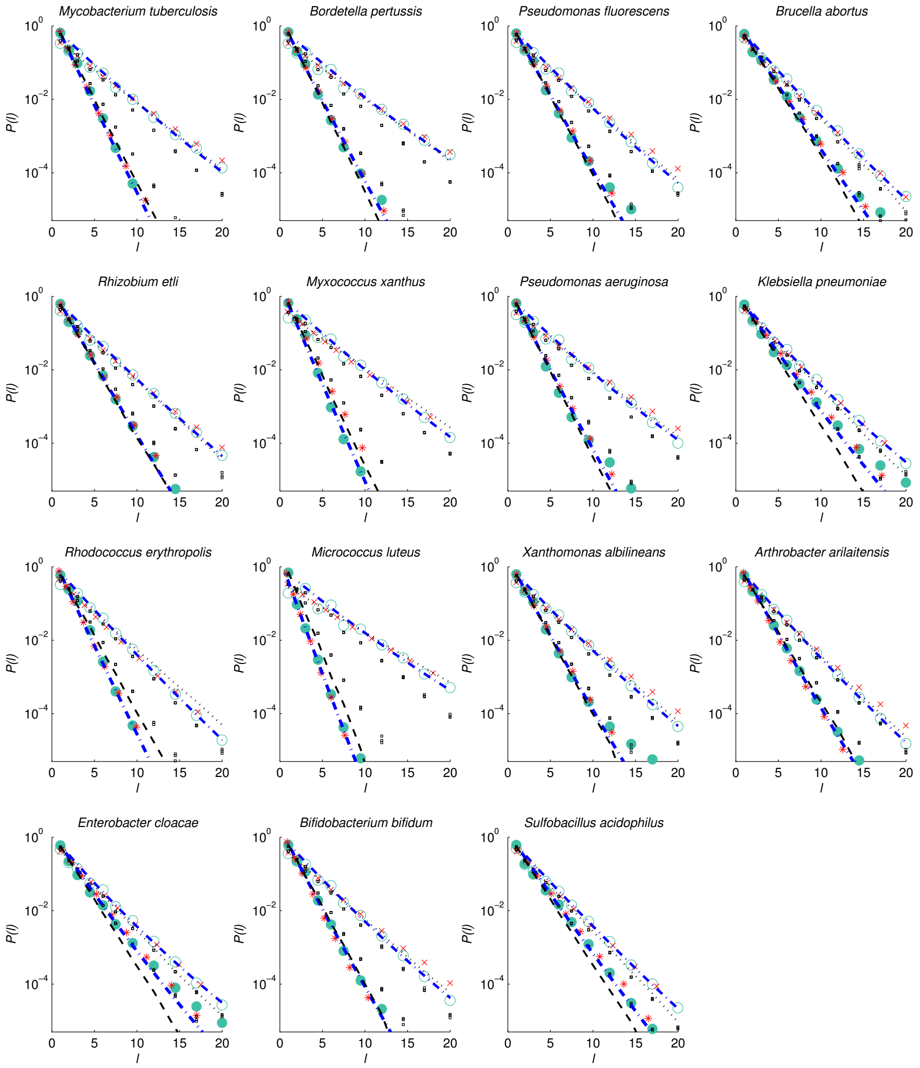

Figure S15: Distribution of the intervals between strongly (•) and weakly (∘) bonded base pairs for bacterial genomes with high GC content. Corresponding model approximations are given by red $*$ for strongly and by red $\times$ for weakly bonded base pairs. Blue dash-dotted lines show corresponding approximations by power law tailed distributions according to Eq. 4. Black dashed and dotted lines show the same distributions for the randomly shuffled DNA sequences for strongly and weakly bonded base pairs, respectively. Small black □ symbols show the same distributions for DNA reconstructed from the corresponding proteome after a randomized back-and-forth translation test.

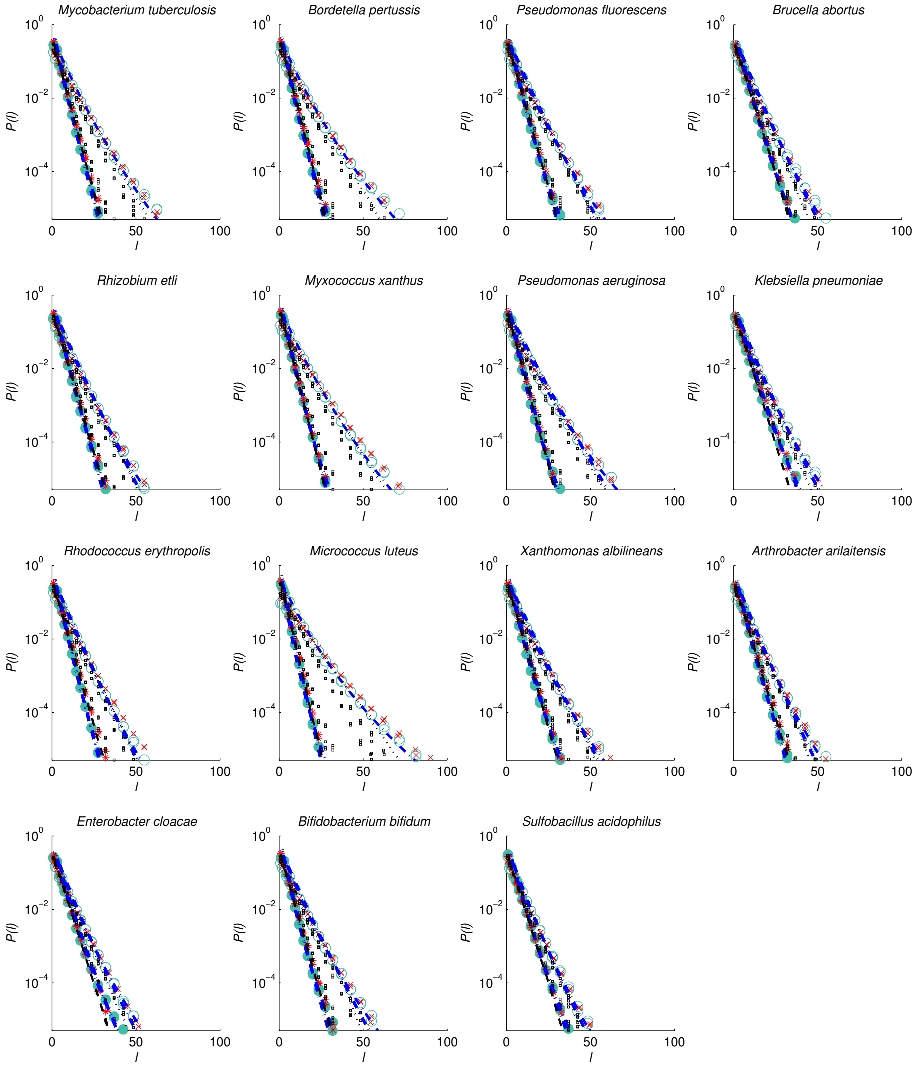

Figure S16: Distribution of the intervals between strongly (•) and weakly (◦) bonded nucleotides for bacterial genomes with high GC content. Corresponding model approximations are given by red $*$ for strongly and by red $\times$ for weakly bonded base pairs. Blue dash-dotted lines show corresponding approximations by power law tailed distributions according to Eq. 4. Black dashed and dotted lines show the same distributions for the randomly shuffled DNA sequences for strongly and weakly bonded nucleotides, respectively. Small black □ symbols show the same distributions for DNA reconstructed from the corresponding proteome after a randomized back-and-forth translation test.

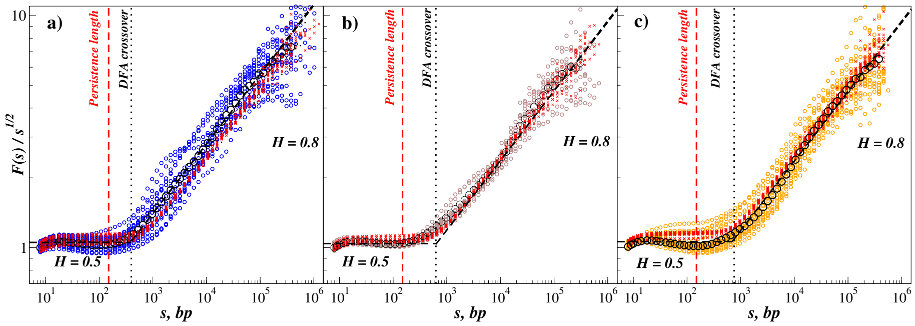

Figure S17: DFA2 fluctuation functions $F(s)$ for the sequence of base pair bond enthalpies for the genomes of extremophile archaeal organisms with optimal living temperatures below 50^oC (left column), between 50^oC and 80^oC (central column) and above 80^oC (right column). Black circles indicate average $F(s)$ within each group, while dashed lines show the approximate model with effectively vanishing correlations below ($H = 0.5$) and pronounced long-range correlations above ($H = 0.8$) the crossover. Corresponding model approximations are given by red $*$. Vertical dotted lines show the position of the crossover, while the red dashed lines indicate the DNA persistence length.

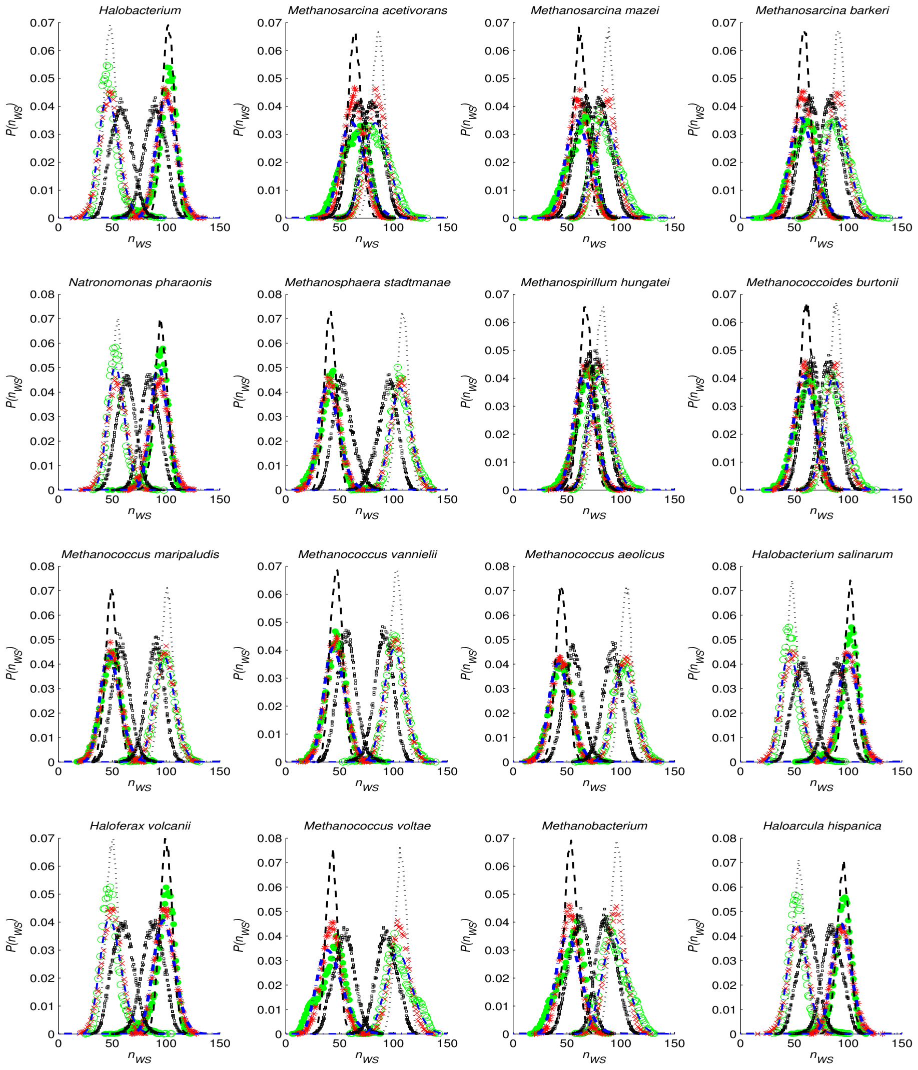

Figure S18: Distribution of the numbers of strongly (•) and weakly (○) bonded base pairs in the local 150 bp DNA segments for genomes of extremophile *Archaea*, with optimal living temperatures below 50^oC. Corresponding model approximations are given by red $*$ for strongly and by red $\times$ for weakly bonded base pairs. Blue dashdot lines show model approximations by Γ-distributions. Black dashed and dotted lines show the same distributions for the randomly shuffled DNA sequences for strongly and weakly bonded base pairs, respectively. Small black □ symbols show the same distributions for DNA reconstructed from the corresponding proteome after a randomized back-and-forth translation test.

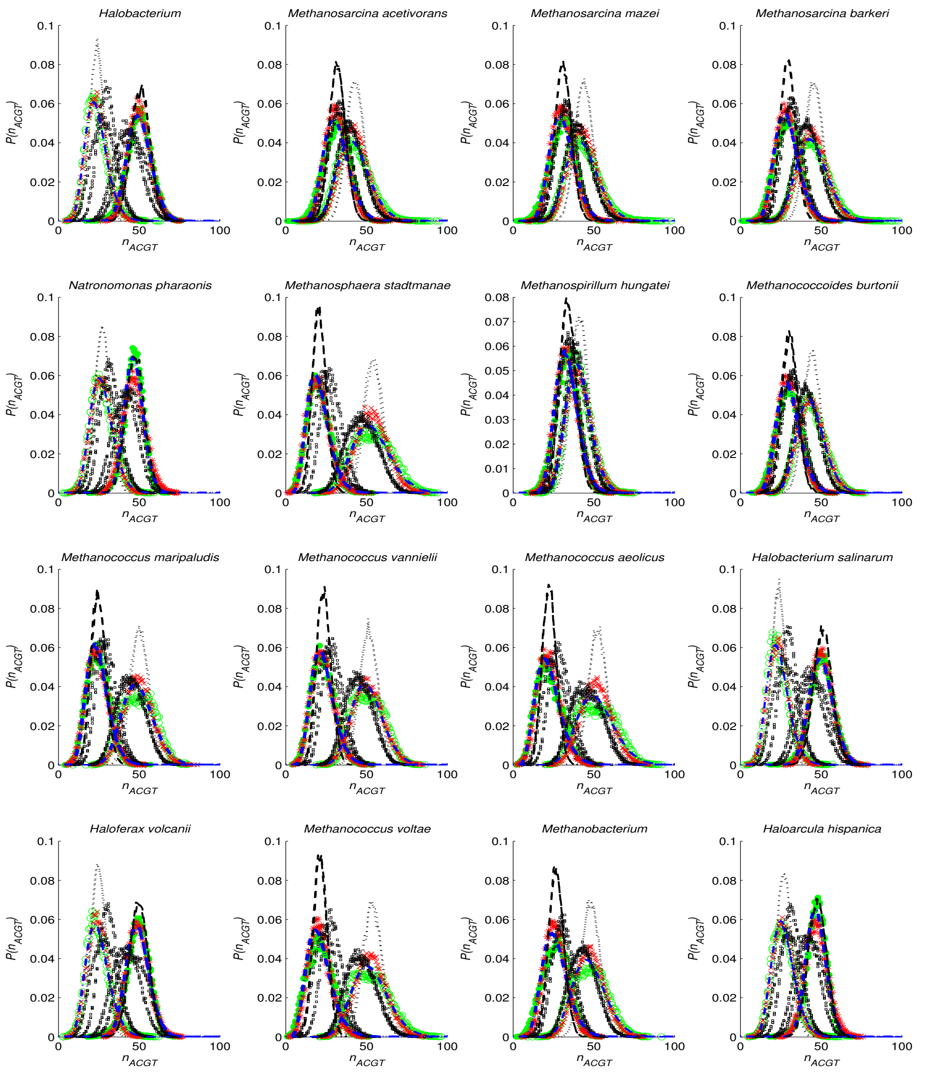

Figure S19: Distribution of the numbers of strongly (•) and weakly (○) bonded nucleotides in the local 150 bp DNA segments for genomes of extremophile *Archaea*, with optimal living temperatures below 50^oC. Corresponding model approximations are given by red $*$ for strongly and by red $\times$ for weakly bonded nucleotides. Blue dashdot lines show model approximations by Γ-distributions. Black dashed and dotted lines show the same distributions for the randomly shuffled DNA sequences for strongly and weakly bonded nucleotides, respectively. Small black □ symbols show the same distributions for DNA reconstructed from the corresponding proteome after a randomized back-and-forth translation test.

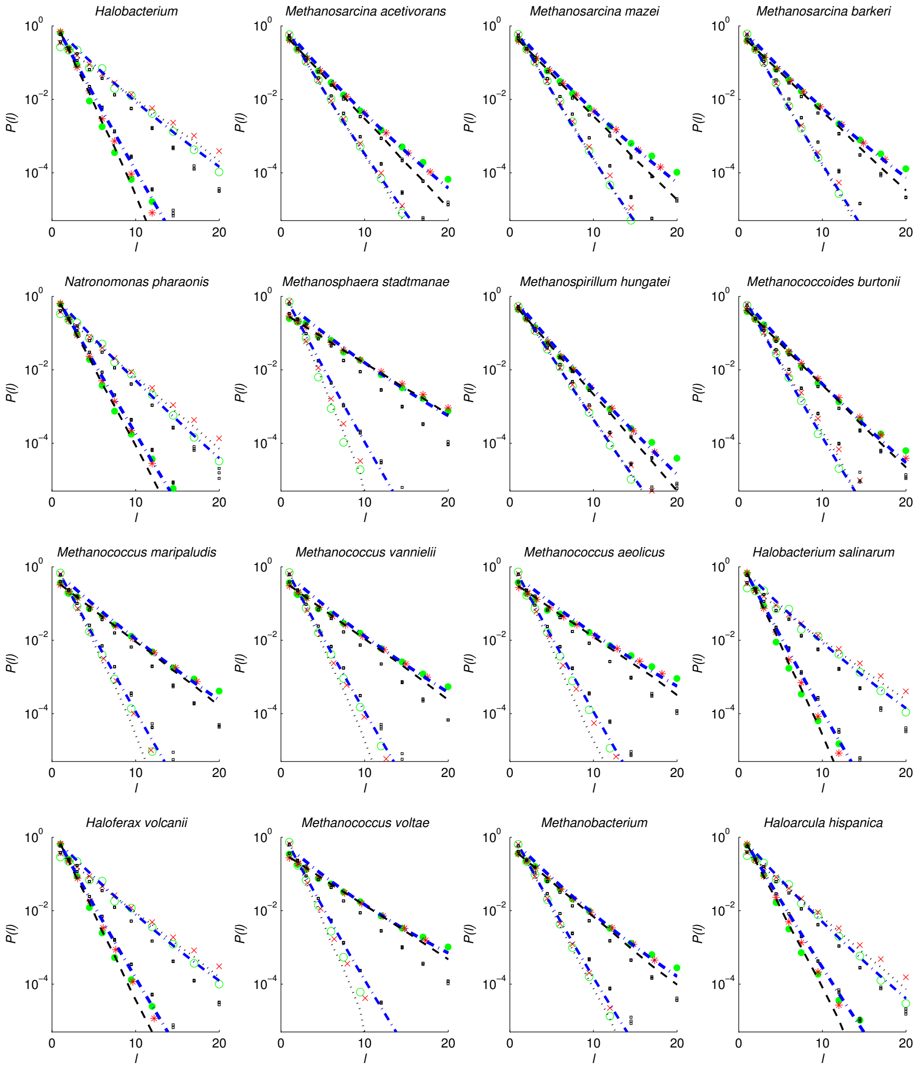

Figure S20: Distribution of the intervals between strongly (•) and weakly (◦) bonded base pairs for genomes of extremophile *Archaea*, with optimal living temperatures below 50^oC. Corresponding model approximations are given by red $*$ for strongly and by red $\times$ for weakly bonded base pairs. Blue dash-dotted lines show corresponding approximations by power law tailed distributions according to Eq. 4. Black dashed and dotted lines show the same distributions for the randomly shuffled DNA sequences for strongly and weakly bonded base pairs, respectively. Small black □ symbols show the same distributions for DNA reconstructed from the corresponding proteome after a randomized back-and-forth translation test.

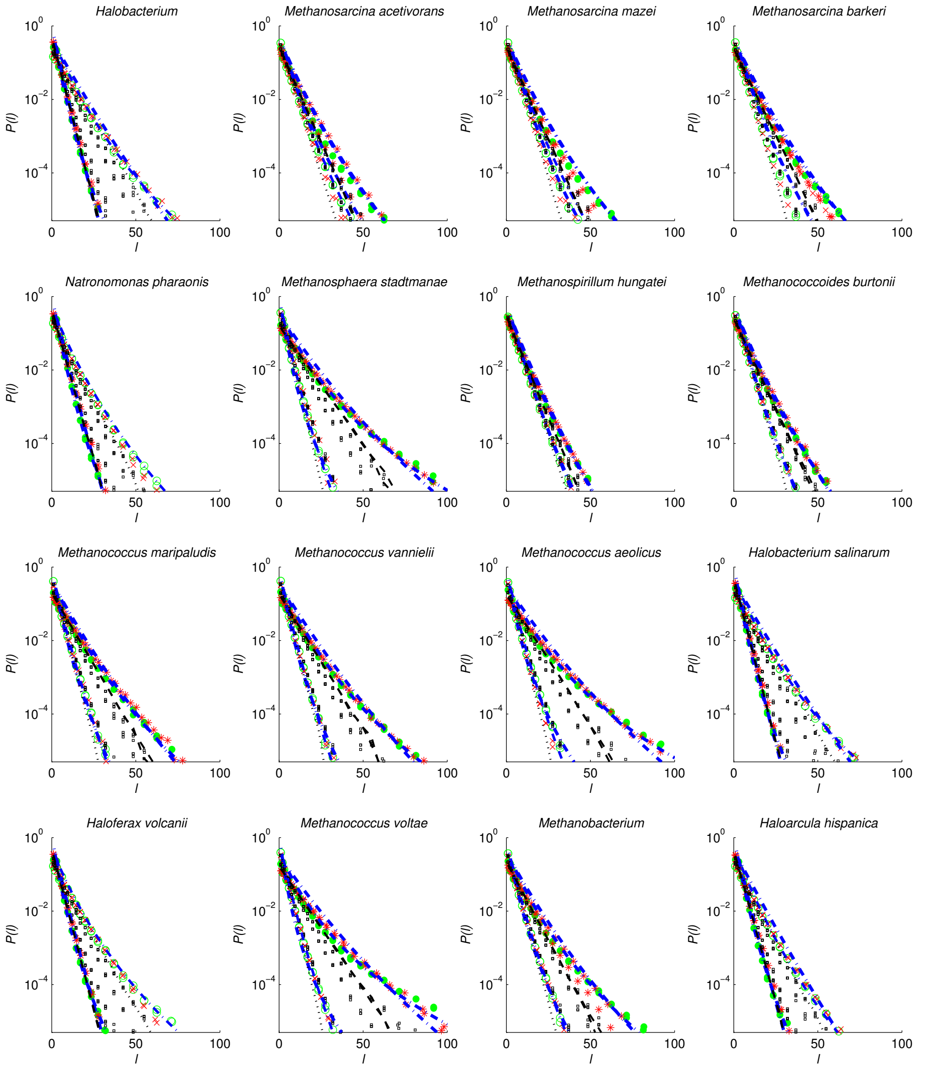

Figure S21: Distribution of the intervals between strongly (•) and weakly (◦) bonded nucleotides for genomes of extremophile *Archaea*, with optimal living temperatures below 50^oC. Corresponding model approximations are given by red ∗ for strongly and by red × for weakly bonded nucleotides. Blue dash-dotted lines show corresponding approximations by power law tailed distributions according to Eq. 4. Black dashed and dotted lines show the same distributions for the randomly shuffled DNA sequences for strongly and weakly bonded nucleotides, respectively. Small black □ symbols show the same distributions for DNA reconstructed from the corresponding proteome after a randomized back-and-forth translation test.

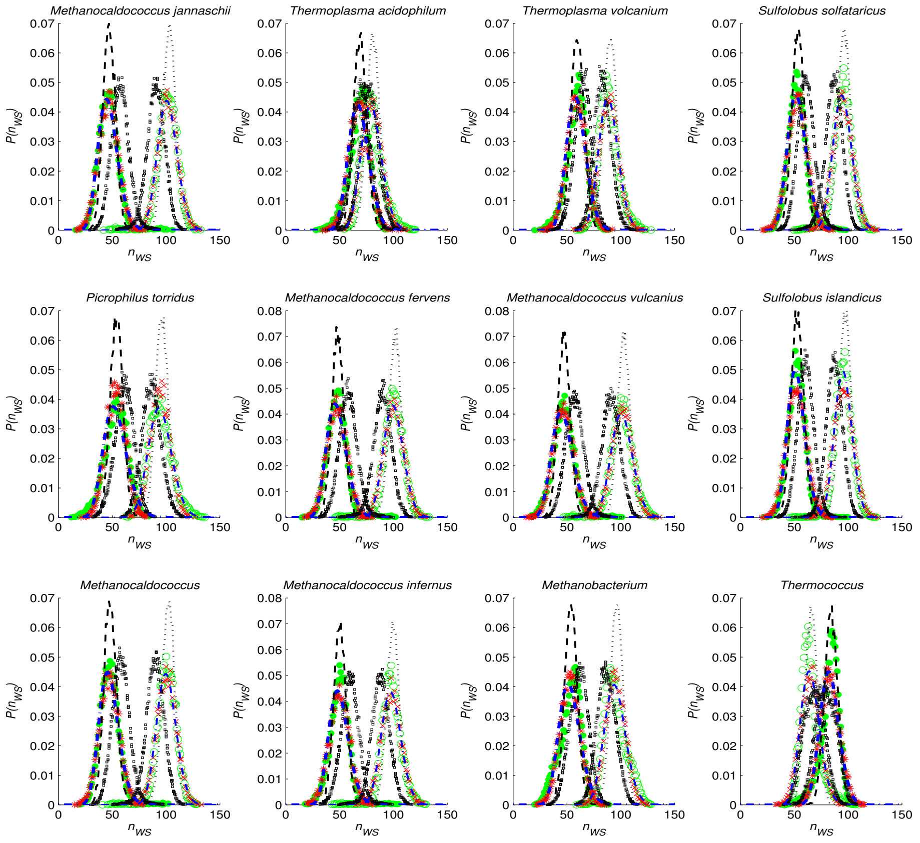

Figure S22: Distribution of the numbers of strongly (•) and weakly (∘) bonded base pairs in the local 150 bp DNA segments for genomes of extremophile *Archaea*, with optimal living temperatures between 50^oC and 80^oC. Corresponding model approximations are given by red $*$ for strongly and by red $\times$ for weakly bonded base pairs. Blue dashdot lines show model approximations by Γ-distributions. Black dashed and dotted lines show the same distributions for the randomly shuffled DNA sequences for strongly and weakly bonded base pairs, respectively. Small black □ symbols show the same distributions for DNA reconstructed from the corresponding proteome after a randomized back-and-forth translation test.

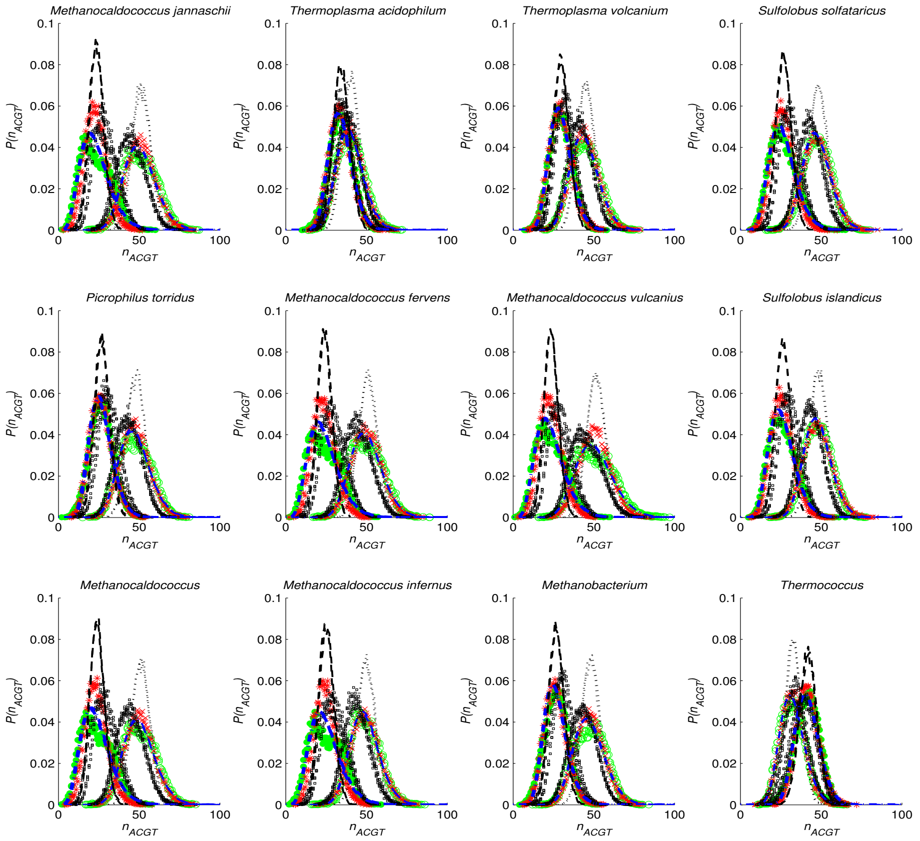

Figure S23: Distribution of the numbers of strongly (•) and weakly (∘) bonded nucleotides in the local 150 bp DNA segments for genomes of extremophile *Archaea*, with optimal living temperatures between 50^oC and 80^oC. Corresponding model approximations are given by red $*$ for strongly and by red $\times$ for weakly bonded nucleotides. Blue dashdot lines show model approximations by Γ-distributions. Black dashed and dotted lines show the same distributions for the randomly shuffled DNA sequences for strongly and weakly bonded nucleotides, respectively. Small black □ symbols show the same distributions for DNA reconstructed from the corresponding proteome after a randomized back-and-forth translation test.

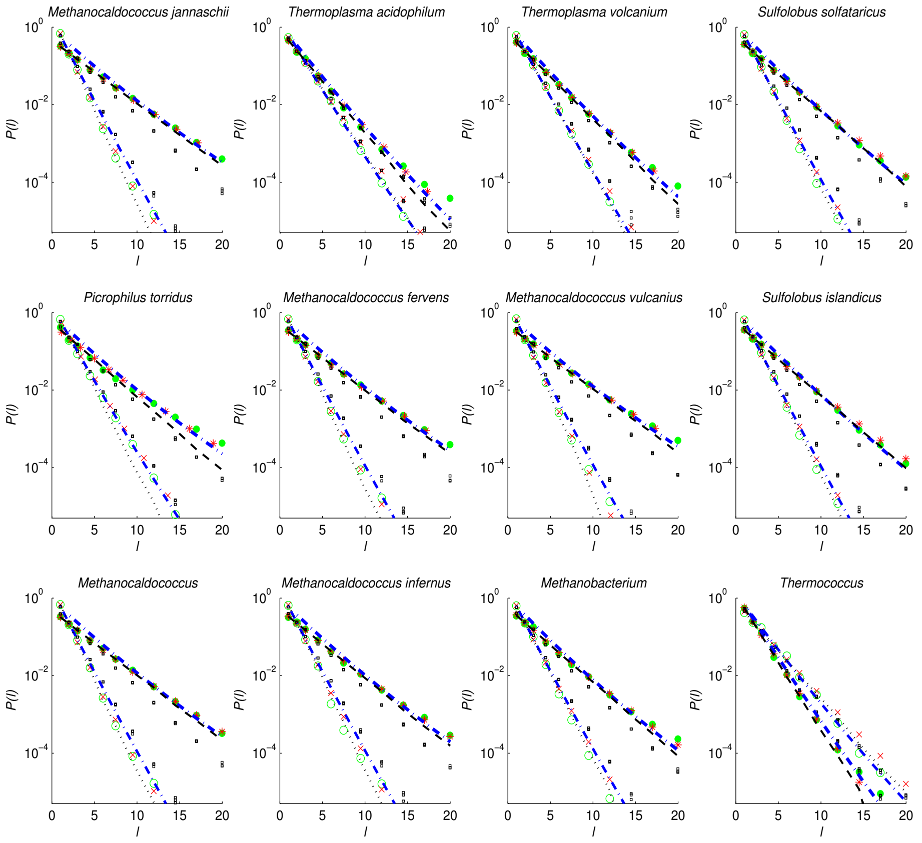

Figure S24: Distribution of the intervals between strongly (•) and weakly (◦) bonded base pairs for genomes of extremophile *Archaea*, with optimal living temperatures between 50^oC and 80^oC. Corresponding model approximations are given by red $*$ for strongly and by red $\times$ for weakly bonded base pairs. Blue dash-dotted lines show corresponding approximations by power law tailed distributions according to Eq. 4. Black dashed and dotted lines show the same distributions for the randomly shuffled DNA sequences for strongly and weakly bonded base pairs, respectively. Small black □ symbols show the same distributions for DNA reconstructed from the corresponding proteome after a randomized back-and-forth translation test.

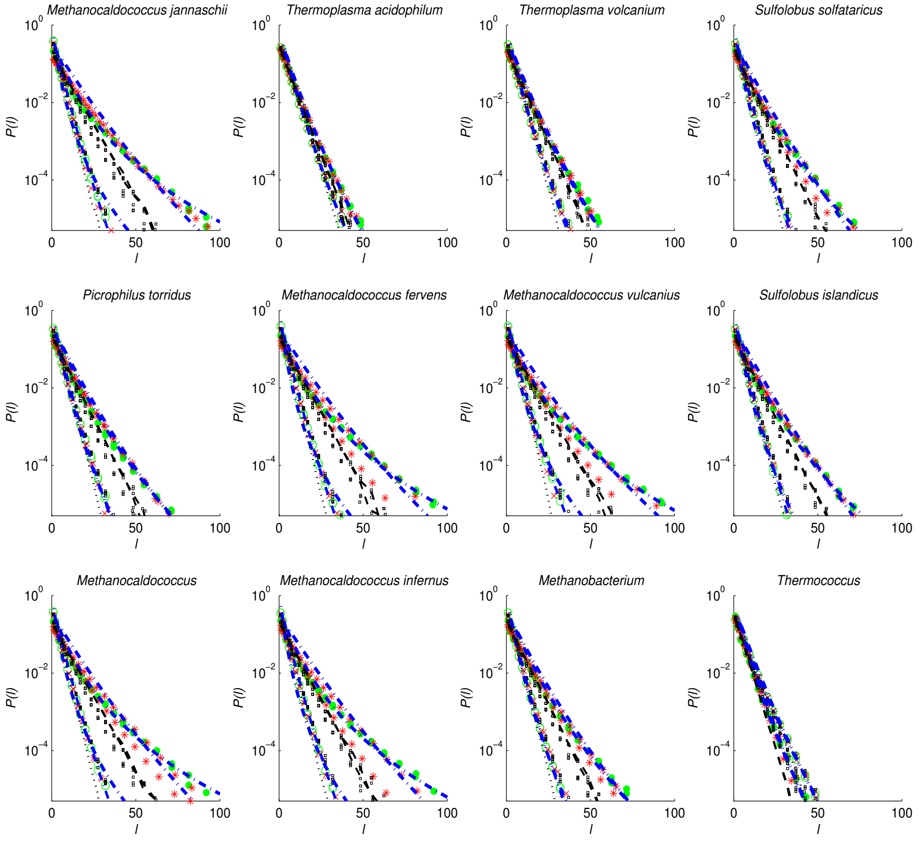

Figure S25: Distribution of the intervals between strongly (•) and weakly (◦) bonded nucleotides for genomes of extremophile *Archaea*, with optimal living temperatures between 50^oC and 80^oC. Corresponding model approximations are given by red $*$ for strongly and by red $\times$ for weakly bonded nucleotides. Blue dash-dotted lines show corresponding approximations by power law tailed distributions according to Eq. 4. Black dashed and dotted lines show the same distributions for the randomly shuffled DNA sequences for strongly and weakly bonded nucleotides, respectively. Small black □ symbols show the same distributions for DNA reconstructed from the corresponding proteome after a randomized back-and-forth translation test.

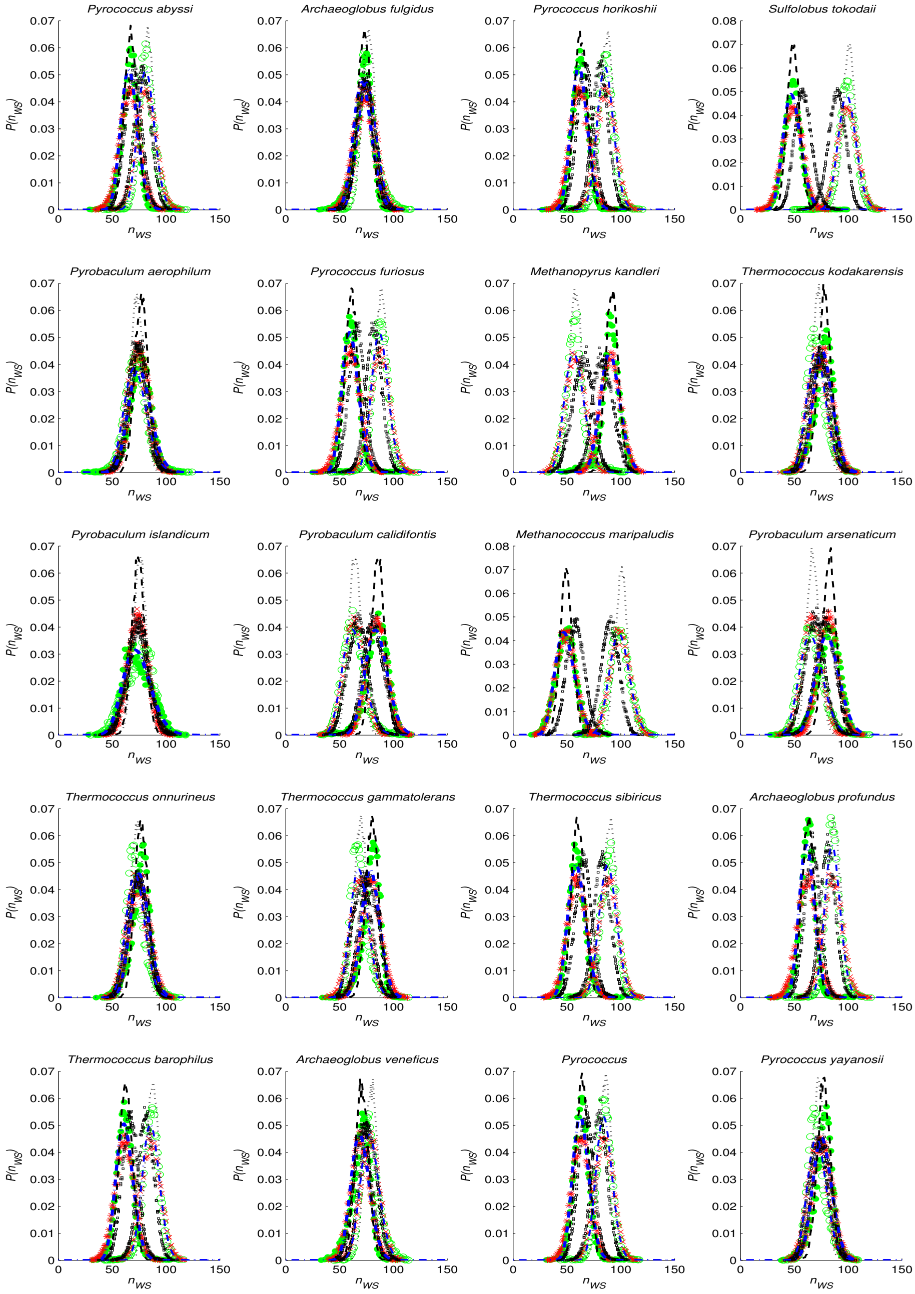

Figure S26: Distribution of the numbers of strongly (•) and weakly (◦) bonded base pairs in the local 150 bp DNA segments for genomes of extremophile *Archaea*, with optimal living temperatures above 80^oC. Corresponding model approximations are given by red $*$ for strongly and by red $\times$ for weakly bonded base pairs. Blue dashdot lines show model approximations by Γ-distributions. Black dashed and dotted lines show the same distributions for the randomly shuffled DNA sequences for strongly and weakly bonded base pairs, respectively. Small black □ symbols show the same distributions for DNA reconstructed from the corresponding proteome after a randomized back-and-forth translation test.

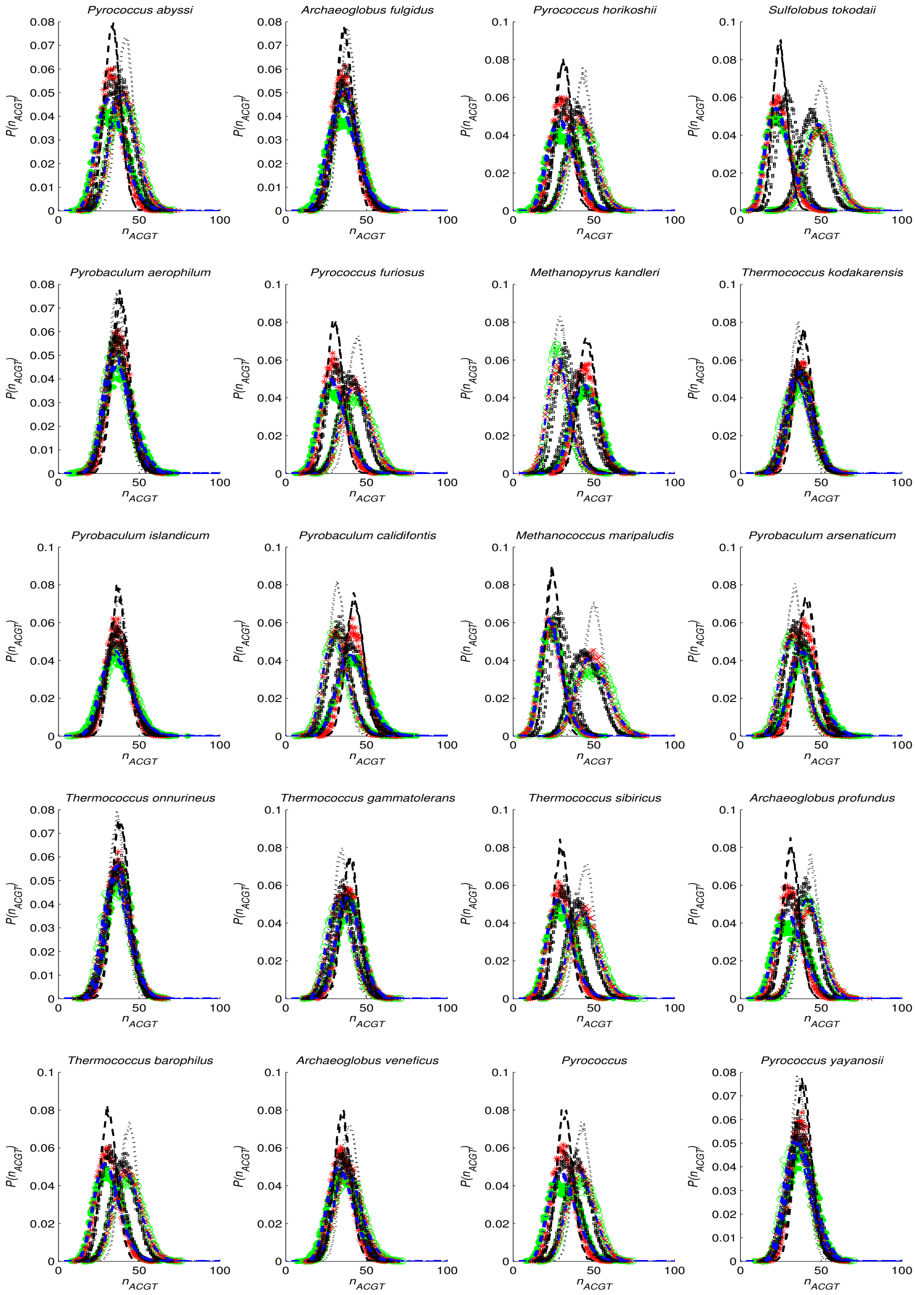

Figure S27: Distribution of the numbers of strongly ($\bullet$) and weakly ($\circ$) bonded nucleotides in the local 150 bp DNA segments for genomes of extremophile *Archaea*, with optimal living temperatures above 80^oC. Corresponding model approximations are given by red $\ast$ for strongly and by red $\times$ for weakly bonded nucleotides. Blue dashdot lines show model approximations by Γ-distributions. Black dashed and dotted lines show the same distributions for the randomly shuffled DNA sequences for strongly and weakly bonded nucleotides, respectively. Small black $\Box$ symbols show the same distributions for DNA reconstructed from the corresponding proteome after a randomized back-and-forth translation test.

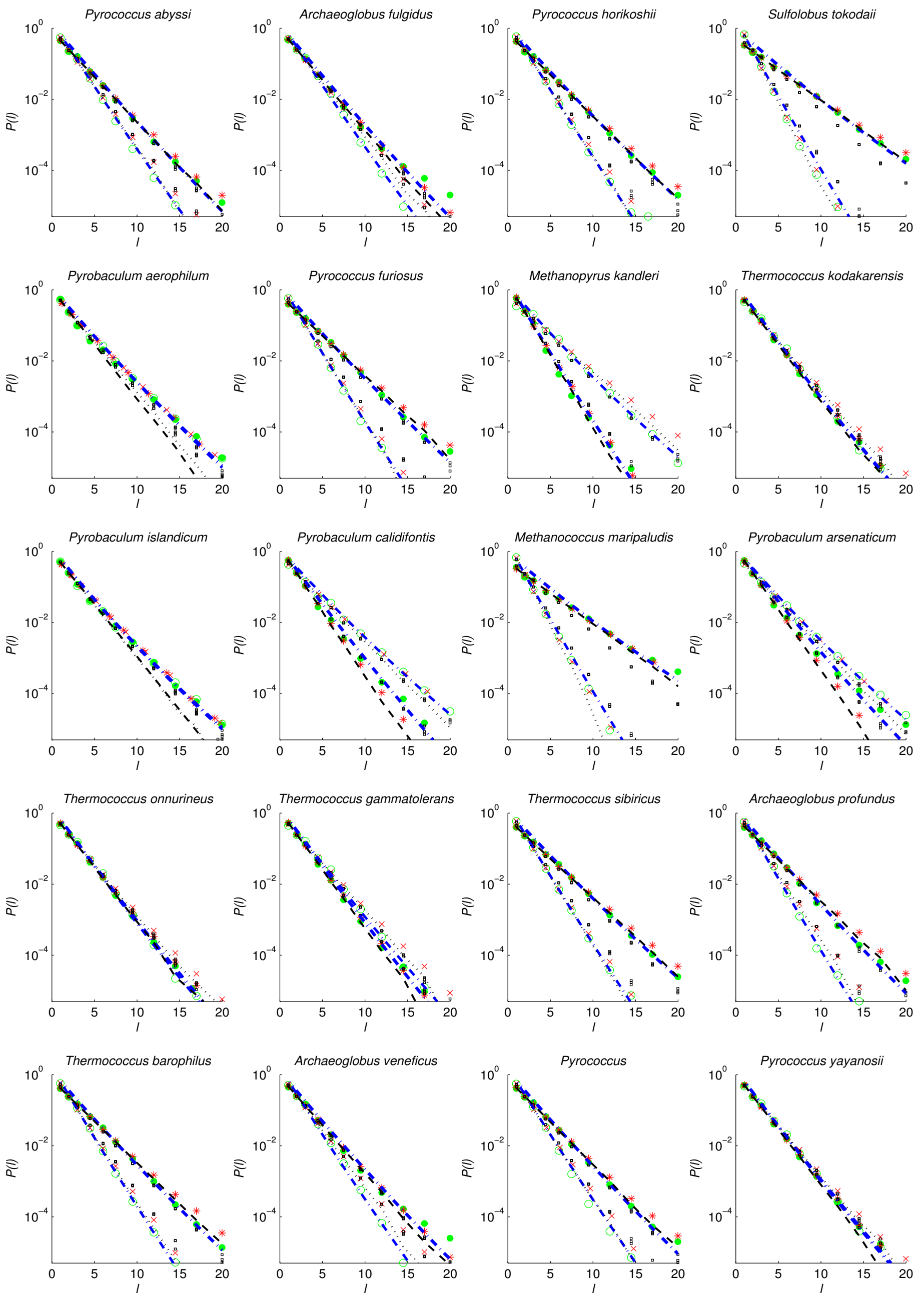

Figure S28: Distribution of the intervals between strongly (•) and weakly (◦) bonded base pairs for genomes of extremophile *Archaea*, with optimal living temperatures above 80^oC. Corresponding model approximations are given by red $*$ for strongly and by red $\times$ for weakly bonded base pairs. Blue dash-dotted lines show corresponding approximations by power law tailed distributions according to Eq. 4. Black dashed and dotted lines show the same distributions for the randomly shuffled DNA sequences for strongly and weakly bonded base pairs, respectively. Small black □ symbols show the same distributions for DNA reconstructed from the corresponding proteome after a randomized back-and-forth translation test.

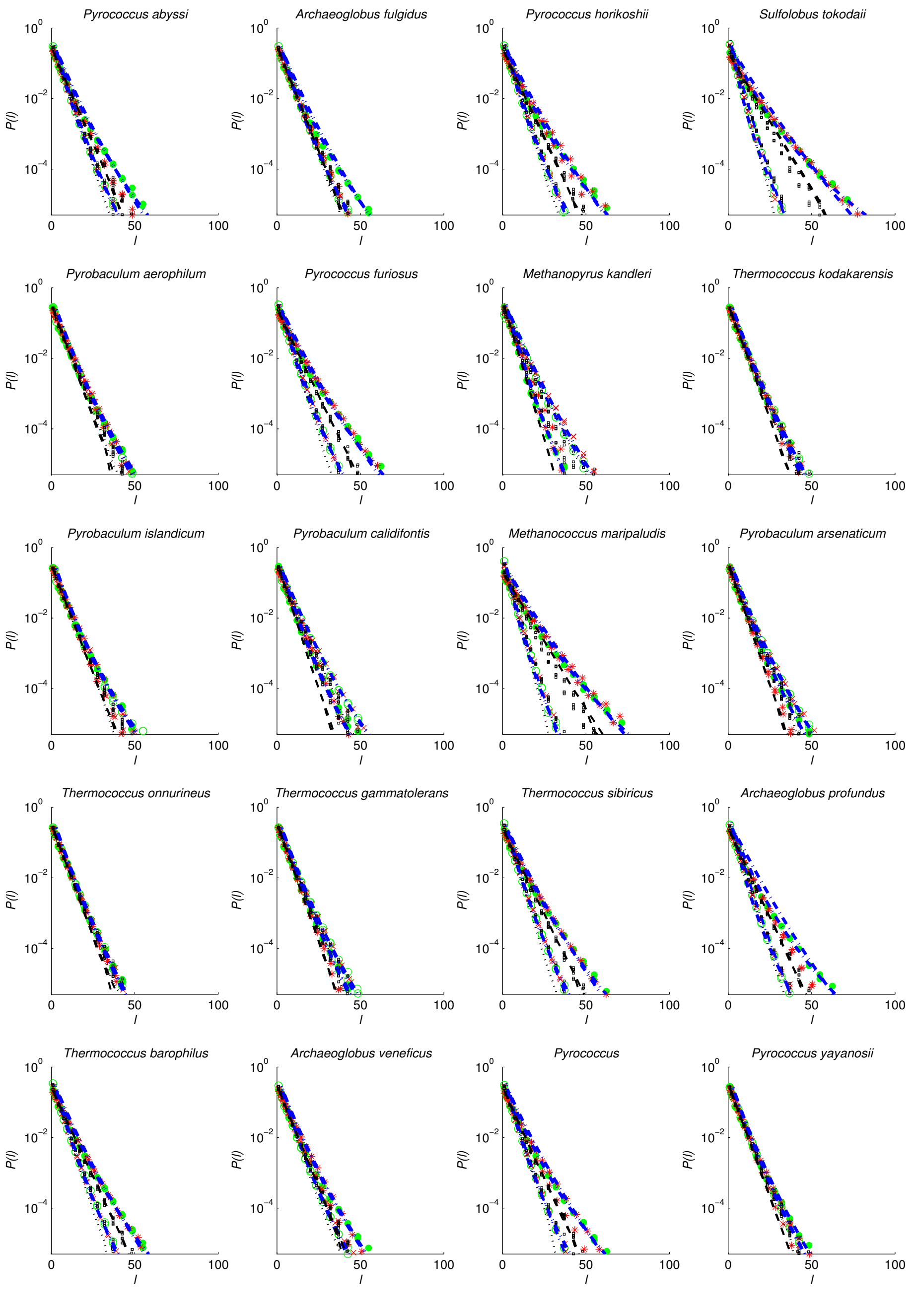

Figure S29: Distribution of the intervals between strongly (•) and weakly (◦) bonded nucleotides for genomes of extremophile *Archaea*, with optimal living temperatures above 80^oC. Corresponding model approximations are given by red $*$ for strongly and by red $\times$ for weakly bonded nucleotides. Blue dash-dotted lines show corresponding approximations by power law tailed distributions according to Eq. 4. Black dashed and dotted lines show the same distributions for the randomly shuffled DNA sequences for strongly and weakly bonded nucleotides, respectively. Small black □ symbols show the same distributions for DNA reconstructed from the corresponding proteome after a randomized back-and-forth translation test.

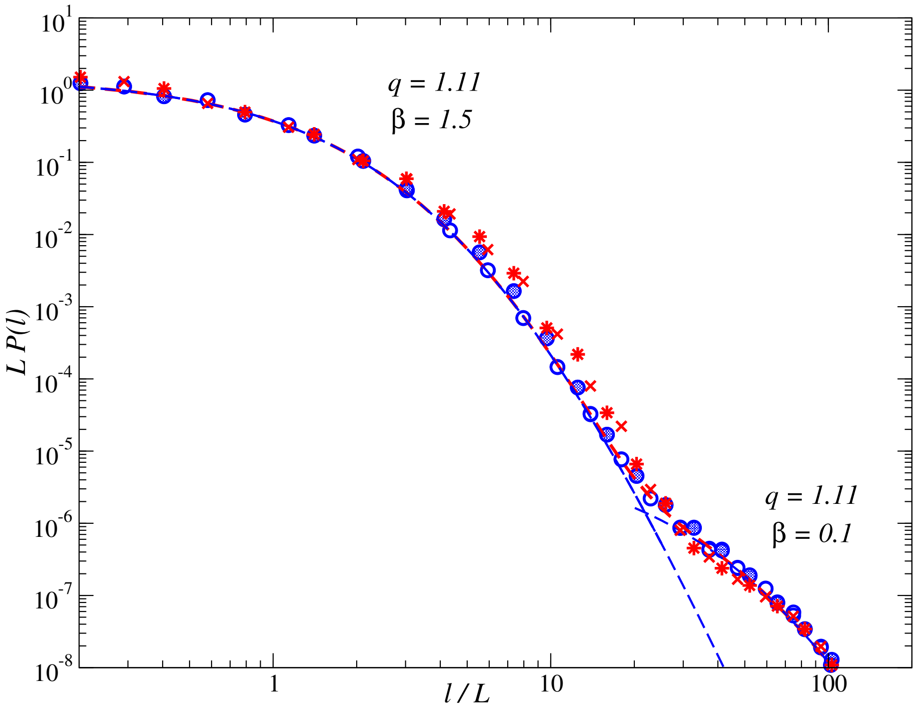

Figure S30: Distribution of the internucleotide intervals in the human genome (full and open circles for strongly and weakly bonded nucleotides, respectively) and its rough approximation by a hierarchical superstatistical model ($*$ and $\times$ for strongly and weakly bonded nucleotides, respectively).
